# Development of a fluorine-18 radiolabelled fluorescent chalcone: evaluated for detecting glycogen

**DOI:** 10.1186/s41181-020-00098-6

**Published:** 2020-06-23

**Authors:** Louis Allott, Diana Brickute, Cen Chen, Marta Braga, Chris Barnes, Ning Wang, Eric O. Aboagye

**Affiliations:** Comprehensive Cancer Imaging Centre, Imperial College London, Hammersmith Hospital, Du Cane Road, London, UK

## Abstract

**Background:**

Glycogen is a multibranched polysaccharide of glucose produced by cells to store energy and plays a key role in cancer. A previously reported fluorescent probe (CDg4) was shown to selectively bind glycogen in mouse embryonic stem cells, however the molecule was not evaluated in cancer cells. We report the synthesis and biological evaluation of a dual-modality imaging probe based on CDg4, for positron emission tomography (PET) and fluorescence microscopy.

**Results:**

A fluorine-18 radiolabelled derivative of CDg4, (**[**^**18**^**F]5**) for in vivo quantification of total glycogen levels in cancer cells was developed and synthesised in 170 min with a non-decay corrected radiochemical yield (RCY n.d.c) of 5.1 ± 0.9% (*n* = 4) in > 98% radiochemical purity. Compound **5** and **[**^**18**^**F]5** were evaluated in vitro for their potential to bind glycogen, but only **5** showed accumulation by fluorescence microscopy. The accumulation of **5** was determined to be specific as fluorescent signal diminished upon the digestion of carbohydrate polymers with α-amylase. PET imaging in non-tumour bearing mice highlighted rapid hepato-biliary-intestinal elimination of **[**^**18**^**F]5** and almost complete metabolic degradation after 60 min in the liver, plasma and urine, confirmed by radioactive metabolite analysis.

**Conclusions:**

Fluorescent compound **5** selectively accumulated in glycogen containing cancer cells, identified by fluorescence microscopy; however, rapid in vivo metabolic degradation precludes further investigation of **[**^**18**^**F]5** as a PET radiopharmaceutical.

## Background

Chalcones are aromatic enones that form the pharmacophore of many biologically important compounds with antitumor, antioxidant and anti-inflammatory properties (Gaonkar and Vignesh 2017; Zhuang et al. 2017). In addition to therapeutic molecules, chalcones have also been investigated as imaging probes, including positron emission tomography (PET) radiotracers targeting beta-amyloid plaques in Alzheimer’s disease (Ono et al. 2010; Chauhan et al. 2014; Ono et al. 2009). The highly conjugated systems can be modified to include electron push-pull pairs to produce fluorescent compounds (Zhuang et al. 2017; Lee et al. 2012). Lee et al. (2012) developed a fluorescent probe based around the chalcone pharmacophore for in vitro imaging of mouse embryonic stem cells (Lee et al. 2012). The lead molecule (CDg4) was identified and hypothesised to bind to the unique secondary structure of glycogen α(1 ➔ 6) in embryonic stem cell colonies. Glycogen is a multibranched polysaccharide of glucose produced by cells to store energy. Glycogen synthesis (glycogenesis) is induced by oncogenic signalling or cellular quiescence and reprogrammed glycogen has been observed in many types of tumours (Zois et al. 2014; Zois and Harris 2016). High glycogen stores have been detected in chemoresistant clear cell adenocarcinoma in ovarian cancer, and is also associated with cancer cells entering the quiescent growth phase (Cheng et al. 2012). Although the mechanisms are not fully understood, the potential for imaging modalities to quantify glycogen storage may identify treatment response biomarkers, thus the development of PET radiopharmaceuticals to measure glycogen levels in tumours via a minimally invasive scan is attractive. We previously reported a radiotracer, [^18^F]*N*-(methyl-(2-fluoroethyl)-1H-[1,2,3]triazole-4-yl) glucosamine ([^18^F]NFTG) for imaging glycogenesis (Carroll et al. 2013). The mechanism of action was based on the enzymatic incorporation of [^18^F] NFTG into the structure of glycogen by glycogen synthase (GS) (Witney et al. 2014). Thus, GS enzyme activity formed the basis of PET signal attributed to [^18^F] NFTG accumulation. A radiopharmaceutical that measures total stored glycogen, rather than GS activity, may provide additional clinical information about disease status in cancer and therefore, the reported CDg4 compound warrants further investigation as a PET probe.

**Fig. 1:**
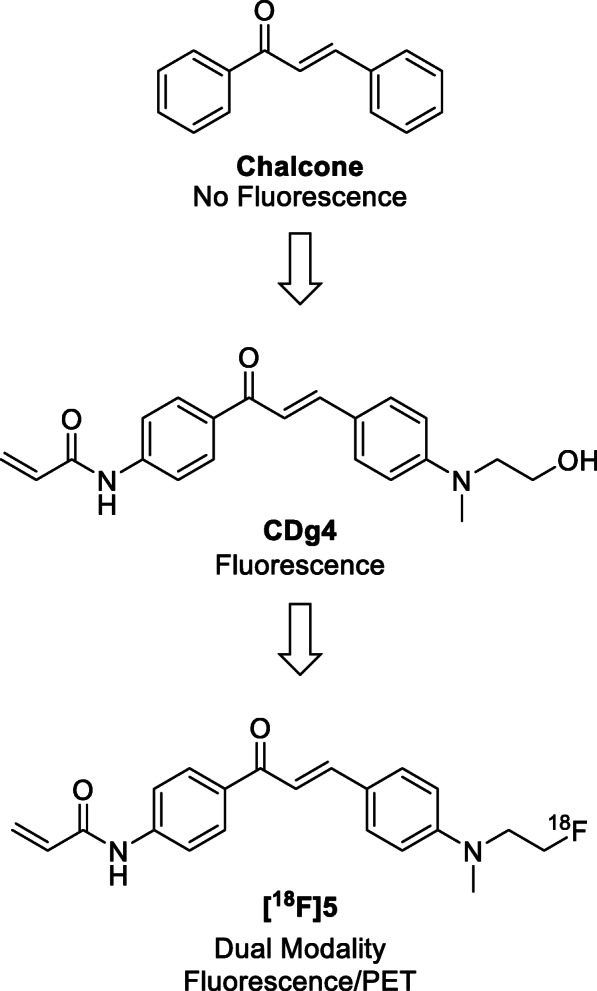
Structural design of a ^18^F-radiolabelled chalcone derivative based on the previously published CDg4 (Lee et al. 2012)

In this work, we propose a dual modality (fluorescence/PET) fluorine-18 radiolabelled molecule based upon the structure of CDg4 that binds to stored glycogen (Fig. 1). Dual-modality probes have the potential to be used in the staging of disease by PET imaging and a tool for fluorescence guided surgery to ensure the effective removal of tumour margins. Despite ubiquitous appearance in biologically important compounds, very few PET probes have been developed around the chalcone pharmacophore and therefore this work provides an opportunity to understand the in vivo biodistribution and metabolism of this interesting class of compounds.

## Results

### Chemistry

**Scheme 1:**
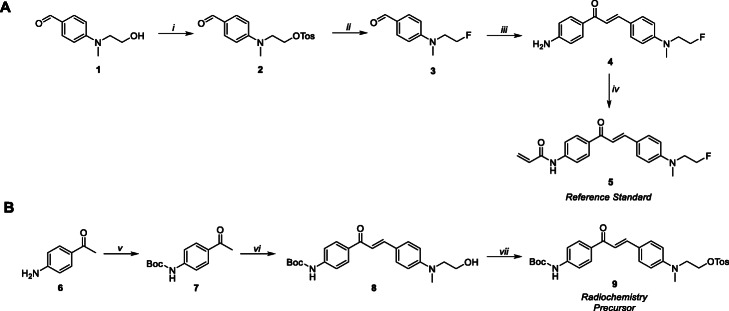
Synthesis of reference standard (**5**) and radiochemistry precursor (**9**). *Reaction conditions:* i) TsCl, TEA, DCM, rt.; ii) TBAF, THF, 100 °C; iii) 4′-aminoacetophenone, NaOH (4 M), EtOH, 50 °C; iv) acryloyl chloride, TEA, CH_3_CN, rt.; v) (Boc)_2_O, TEA, DCM, rt.; vi) *tert*-butyl (4-acetylphenyl) carbamate, NaOH, EtOH, 50 °C; vii) TsCl, pyridine, 4 h at 0 °C and then rt

Fluorescent probe **5** (Scheme 1a) was synthesised along with a precursor **9** (Scheme 1b) for the radiosynthesis of **[**^**18**^**F]5**. To access compound **5**, tosylation of the hydroxyl group on compound **1** gave compound **2**. Fluorination of intermediate **2** with tert-butylammonium fluoride (TBAF) furnished compound **3** in an excellent yield of 97%. Compound **3** was reacted with 4′-aminoacetophenone (**6**) in an aldol condensation reaction to give chalcone **4** which was challenging to purify and resulted in a low yield (16%). Structural identity of the *E*-isomer was confirmed using NMR by their characteristic J-values (15.4 Hz). Compound **5** was produced by reacting acryloyl chloride with **4** to install the terminal acrylamide moiety. The photochemical properties (excitation: λ_ex_ and emission: λ_em_) of **5** were evaluated by UV-Vis and fluorescence spectroscopy (ESI, Figure S18) and found to be λ_ex_ = 420 nm / λ_em_ = 550 nm. The radiochemistry precursor **9** was synthesised in three steps (Scheme 1b). Firstly, the amine of 4′-aminoacetophenone (**6**) was boc-protected (**7**) and reacted with 4-((2-hydroxyethyl)(methyl)amino) benzaldehyde to yield chalcone **8** (30% yield). The final radiochemistry precursor was produced by converting the hydroxyl moiety into a tosylate leaving group (**9**) for subsequent radiochemistry development. All compounds were characterised by ^1^H-NMR, ^13^C-NMR, ^19^F-NMR and mass spectrometry (ESI, Figure S1–17).

### Radiochemistry

**Scheme 2:**
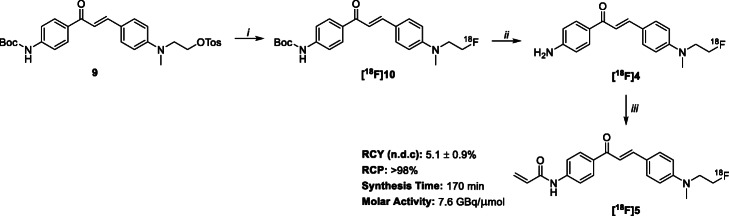
Radiochemistry to access **[**^**18**^**F]5**. *Reaction conditions:* i) [^18^F] fluoride, Kryptofix K_222_, KHCO_3_, MeCN, 90 °C, 20 min; ii) phosphoric acid (1.8 M), MeCN, 80 °C, 15 min; iii) acryloyl chloride, MeCN, 50 °C, 15 min

The fluorine-18 radiolabelled chalcone (**[**^**18**^**F]5**) was synthesised in a three-step radiosynthesis and is fully described in the Methods section and ESI. Compound **[**^**18**^**F]5** was produced in a non-decay corrected radiochemical yield (RCY n.d.c) of 5.1 ± 0.9% (*n* = 4) in 170 min with a molar activity of 7.6 GBq/μmol. The LogD_7.5_ of **[**^**18**^**F]5** was determined experimentally using the shake-flask method and found to be 1.03 ± 0.37 (Scheme 2).

### Fluorescence microscopy

**Fig. 2:**
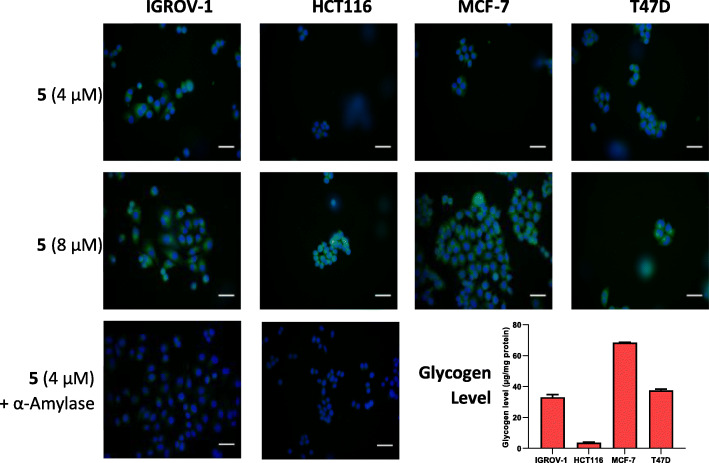
Fluorescence microscopy of compound **5** (green fluorescence), nuclear staining (blue fluorescence) in four cancer cell lines. Compound **5** was also incubated with α-amylase in IGROV-1 (high glycogen content) and HCT116 (low glycogen content) cells to digest carbohydrates. Images were obtained under × 400 magnification. Scale bar = 50 μm. Inset graph: Total intracellular glycogen levels determined by a modified glucose (GO) assay kit. Cells were seeded in 6-well plates at appropriate densities and allowed to attach overnight before determination of glycogen levels. Data are mean ± SD (*n* = 3)

Compound **5** was evaluated by fluorescence microscopy in a panel of four cancer cell lines with differential glycogen expression (IGROV-1, HCT116, MCF-7 and T47D). Cells showed a concentration-dependent (with respect to compound **5**) accumulation of fluorescence (Fig. 2) and specificity of binding was inferred by decreased fluorescence when compound **5** was incubated in cells treated with α-amylase to digest carbohydrate polymers. These data suggest a concentration-dependent accumulation of **5**, specific to glycogen.

### In vitro cell uptake of [^18^F]5

**Fig. 3:**
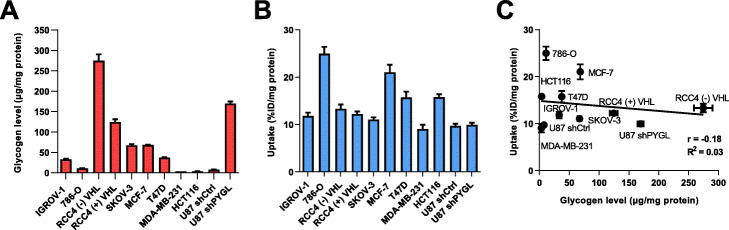
Correlation of **[**^**18**^**F]5** uptake to total glycogen level among a panel of cancer cell lines. **a** Total intracellular glycogen levels determined by a modified glucose (GO) assay kit. Cells were seeded in 6-well plates at appropriate densities and allowed to attach overnight before determination of glycogen levels. Data are mean ± SD (*n* = 3); **b** In vitro uptake of **[**^**18**^**F]5**. Cells under the same culturing conditions were incubated with fresh DMEM media containing 0.74 MBq **[**^**18**^**F]5** for 1 h at 37 °C in a humidified atmosphere of 5% CO_2_. Data are mean ± SD (*n* = 6), expressed as % of incubated radioactive dose, normalized to protein content (%ID/mg). **c** Correlation between total glycogen levels and **[**^**18**^**F]5** uptake. The best-fit line is shown with a correlation coefficient (*r*) = − 0.18 or *R*^*2*^ = 0.03

The total glycogen level was determined in a panel of cancer cell lines using a modified GO assay kit (Fig. 3a). The in vitro uptake of **[**^**18**^**F]5** was evaluated in the same cell lines (Fig. 3b). The average uptake of **[**^**18**^**F]5** was 11.01 ± 0.62% of incubated activity dose/mg of total protein (%ID/mg) in the cell lines with lowest uptake, and even higher for 786-O, MCF7, T47D and HCT116, where uptake was 1.4–2.3 fold higher (24.98 ± 1.43, 21.02 ± 1.61, 15.73 ± 1.23 and 15.80 ± 0.61%ID/mg, respectively). It was expected that **[**^**18**^**F]5** uptake would correlate with total glycogen level however, despite stark differences in uptake in some cell lines, no correlation (R^2^ = 0.03) with total glycogen was observed (Fig. 3c).

### PET imaging

**Fig. 4:**
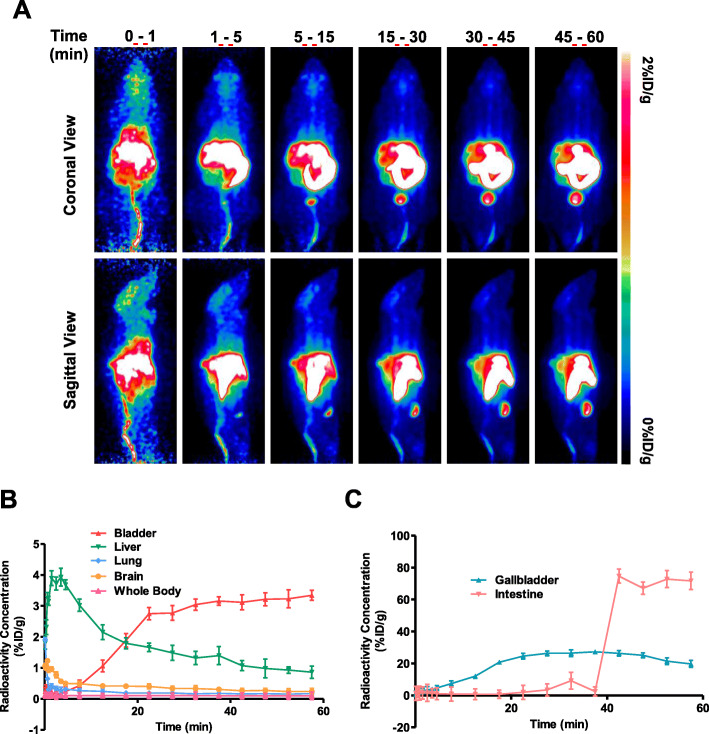
PET imaging of **[**^**18**^**F]5**. **a** Representative maximum intensity projection PET images of BALB/c mice following injection of 1.48 MBq of **[**^**18**^**F]5** via tail vein. Time frames were summed into images representing 0–1, 1–5, 5–15, 15–30, 30–45 and 45–60 min post radioactive injection. Coronal (top) and sagittal (bottom) projections are shown. **b** Time activity curve of **[**^**18**^**F]5** in bladder, liver, lung, brain and whole body over 60 min post i.v. injection of 1.48 MBq of **[**^**18**^**F]5** via tail vein, expressed as % of injected dose normalised to body weight (%ID/g). **c** Same as **b** but in gallbladder and intestine. Data are mean + SEM, *n* = 4

PET imaging was used to determine pharmacokinetics (PK), biodistribution and in vivo metabolism of **[**^**18**^**F]5** in four non-tumour bearing mice, to maximise the quantity of useful data from as few animals as possible. The PK and biodistribution of **[**^**18**^**F]5** in normal BALB/c mice were evaluated in vivo using dynamic PET imaging (Fig. 4a). **[**^**18**^**F]5** accumulated rapidly in the intestine, gallbladder and eventually bladder (> 30 min p.i.) (Fig. 4b, c); although high levels of radioactivity were initially seen in the liver (0–10 min p.i.), a subsequent fast wash-out suggests efflux or fast metabolism of the tracer rather than non-specific accumulation. Put together, these data suggest rapid metabolism and clearance through hepato-biliary-intestinal route.

### In vivo metabolite analysis

**Fig. 5:**
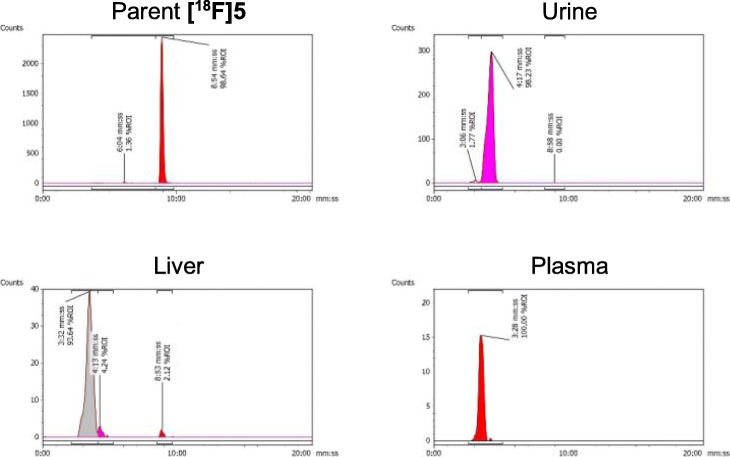
Representative radio-HPLC chromatogram of parent **[**^**18**^**F]5** (t_R_ = 08:54 min:sec) and metabolites in urine, liver and plasma at 60 min p.i.; extraction efficiencies of radioactivity from plasma and liver were 73.7 ± 2.5 and 45.2 ± 4.3% total radioactivity, respectively

The in vivo metabolic stability of **[**^**18**^**F]5** was determined in liver, plasma and urine by analytical radio-HPLC at 60 min p.i. A single polar radioactive metabolite (> 99%) was observed in all measured samples except for liver where < 3% of parent **[**^**18**^**F]5** remained (Fig. 5).

## Discussion

To develop a dual fluorescence/PET imaging probe based on the structure of CDg4, three important properties were considered:

i) Target recognition - maintaining the pharmacophore of CDg4 to ensure a fluorine-18 radiolabelled derivative would have similar biological properties.
ii) Fluorescence - retention of the push-pull electron system to maintain similar fluorescence properties to CDg4.
iii) PET radionuclide - incorporation of a fluorine atom into a synthetically accessible location to allow for simple fluorine-18 radiochemistry.

To satisfy all three properties, the terminal hydroxyl group of CDg4 was selected as a suitable location to incorporate a fluorine atom. This modification avoided change to the chalcone core structure and was hypothesised to retain similar glycogen binding properties as CDg4. Lee et al. (2012) had previously incorporated this hydroxyl moiety into a library of 160 compounds analogous to CDg4, to improve the hydrophilicity of the molecules and for tethering to a solid-support for the simple synthesis of the large library; the hydroxyl was deemed non-essential for binding glycogen as only CDg4 uptake corelated with glycogen despite all compounds bearing the hydroxyl moiety. Modification of CDg4 distant to the conjugated aromatic system of the chalcone pharmacophore was expected to exhibit minimal perturbation of the existing electronic properties of the molecule, therefore similar fluorescent properties would be retained. Exchanging the hydroxyl group for fluorine at this position would allow for a dual fluorescence/PET probe to be synthesised from simple radiochemistry methods.

Compound **5**, a fluorine-containing derivative of CDg4, was synthesised in four steps and was used as a fluorescent probe for visualising glycogen by fluorescence microscopy and as a HPLC reference standard to confirm the identity of the fluorine-18 radiolabelled analogue (**[**^**18**^**F]5**). Compound **5** and CDg4 exhibited similarity in photochemical properties, determined by UV-Vis and fluorescence spectroscopy, which inferred that replacing the hydroxyl group with fluorine had minimal impact on the electron push-pull pairs of the molecule (CDg4: λ_ex_ = 430 nm, λ_em_ = 560 nm; **5**: λ_ex_ = 420 nm / λ_em_ = 550 nm); therefore, we also expect the quantum yield to be similar to CDg4 (Φ = 0.2). The λ_ex_ and λ_em_ wavelengths were used for the biological evaluation of **5** by fluorescence microscopy.

To access the fluorine-18 radiolabelled compound **[**^**18**^**F]5**, a radiochemistry precursor was synthesised. The terminal hydroxyl moiety of compound **8** was converted into a good leaving group by tosylation, producing a boc-protected precursor (**9**) for simple S_N_2 displacement with [^18^F]fluoride. Producing **[**^**18**^**F]5** in a single-step from an appropriate radiochemistry precursor was avoided as the acryloyl group was identified as potentially sensitive to the basic conditions and high temperatures required for the fluorine-18 radiochemistry. The boc-protected precursor (**9**) allowed effective radiolabelling and installation of the acryloyl moiety post-fluorination in a three-step radiosynthesis. In brief, **[**^**18**^**F]10** was synthesised from precursor **9** via nucleophilic substitution (S_N_2) by displacement of the tosylate leaving group with anhydrous [^18^F]fluoride. Intermediate **[**^**18**^**F]10** was purified from unreacted [^18^F] fluoride using a tC18 solid-phase extraction (SPE) cartridge and eluted in acetonitrile for further reactions. To reduce radiation exposure, the drying of [^18^F] fluoride and the first radiolabelling step was automated using the GE FASTlab™ platform (ESI, Figure S19), which allowed larger starting activities (4–6 GBq) of [^18^F] fluoride to be used. Hydrolysis of the protecting group was achieved under acidic conditions and the resulting compound **[**^**18**^**F]4** was trapped on a tC18 SPE cartridge and dried under a vigorous flow of nitrogen. Thorough drying of intermediate **[**^**18**^**F]4** was necessary to ensure an efficient reaction with to acryloyl chloride in the final step of the radiosynthesis, producing compound **[**^**18**^**F]5**. The final radioconjugate **[**^**18**^**F]5** was purified by semi-preparative HPLC and the desired fraction was reformulated into ethanol by tC18 SPE cartridge for biological evaluation. The identity of **[**^**18**^**F]5** was confirmed by HPLC analysis by co-injection with authentic non-radioactive reference standard **5** and the radiochemical purity (RCP) was > 98%, produced in an acceptable RCY (n.d.c) of 5.1 ± 0.9% and molar activity (A_m_ = 7.6 GBq/μmol).

The cellular uptake of compound **5** and **[**^**18**^**F]5** was evaluated in vitro by fluorescence microscopy and PET to determine their ability to bind glycogen in cell-based studies. The in vivo biodistribution and metabolic stability of **[**^**18**^**F]5** was evaluated in mice and was conducted with the intention of identifying a promising molecule for preclinical evaluation, and further, clinical translation into humans.

The levels of glycogen in a panel of 11 cancer cell lines was determined experimentally for this study (Fig. 3a). From this panel a subset of four cell lines where glycogen expression was not upregulated by genetic manipulation were selected to evaluate the ability of **5** to bind glycogen determined by fluorescence microscopy; three of which (IGROV-1, MCF-7 and T47D) exhibited higher glycogen storage compared to HCT116 which was used as a negative control. As our interests were predominantly in developing a glycogen targeted PET probe, only **[**^**18**^**F]5** was evaluated in all 11 cancer cell lines. Compound **5** accumulated in cells in a concentration-dependent manner, and the fluorescent signal was diminished when the cells were treated with α-amylase to digest carbohydrate polymers, suggesting the accumulation of **5** was specific to glycogen. The lowest fluorescence intensity was observed in HCT116 cells which was consistent with the measured glycogen levels. This data supports the observations made by Lee et al. (2012) for CDg4; quantification of glycogen levels by fluorescence was not investigated as PET, a more sensitive quantification technique, would be performed with compound **[**^**18**^**F]5**.

The in vitro uptake of radiolabelled **[**^**18**^**F]5** was performed in all 11 cancer cell lines for correlation to experimentally determined glycogen levels (Fig. 3). The average uptake of **[**^**18**^**F]5** was 11.01 ± 0.62%ID/mg across all cell lines, however some showed 1.4–2.3 fold higher accumulation; no correlation with total glycogen levels was observed (Fig. 3c). It was hypothesised that the mechanism of uptake for **[**^**18**^**F]5** was likely to be via passive diffusion due to its slightly lipophilic structure (LogD_7.5_ = 1.03 ± 0.37) and that the retention of **[**^**18**^**F]5** in cells, and the large difference in uptake between cell lines (1.4–2.3 fold difference), may be due to non-specific interactions; perhaps facilitated by the formation of covalent bonds between **[**^**18**^**F]5** and cellular components (e.g. proteins, biomolecules) through the acrylamide moiety, a potent Michael acceptor. This functional group has been used in the design of covalent irreversible receptor inhibitors.

Despite promising fluorescence microscopy data, which suggested that the accumulation of **5** in cells was specific to a carbohydrate polymer, the evaluation of **[**^**18**^**F]5** did not yield comparable results. The difference in the in vitro performance of **5** and **[**^**18**^**F]5** may have resulted from the so-called “mass effect”; the quantity of fluorescent probe **5** (lowest at 4 μM) was 40-fold larger in comparison to PET probe **[**^**18**^**F]5** present at ca 100 nM (determined from A_m_), which may have been sufficient to overcome non-specific reactivity towards cellular components. The use of **[**^**18**^**F]5** at lower A_m_ (0.18 GBq/μmol, equivalent of 4 μM) to replicate the mass of compound used in fluorescent experiments was not performed as the translation of low molar activity tracers into clinical imaging is not routine. A similar phenomenon has been exemplified for covalent epidermal growth factor receptor (EGFR) inhibitors which, despite many being permeability glycoprotein (PGP) substrates, are still effective drugs when high doses are administered (Xu and Li 2019, Floc’h et al 2018, Waring et al 2018). This hypothesis is perhaps corroborated by the unusually high uptake of **[**^**18**^**F]5** across all cell lines (ca. 10–25%ID/mg), suggesting that retention within cells was based on a non-specific mechanism.

Although **[**^**18**^**F]5** showed no correlation to cellular glycogen, the high uptake warranted further investigation into the PK, biodistribution and metabolic fate of this molecule. This understanding may inform the design of new **[**^**18**^**F]5** analogues that effectively bind glycogen and are suitable candidates for pre-clinical and clinical translation. Compound **[**^**18**^**F]5** rapidly accumulated in the liver within 10 min p.i. followed by the intestines, gallbladder and eventually the bladder after 30 min p.i. suggesting hepato-biliary-intestinal clearance. Rapid clearance is advantageous for tumour imaging by improving the signal-to-background ratio, provided the tumour is not located in the abdominal region.

Metabolite analysis of **[**^**18**^**F]5** in the plasma, liver and urine showed almost complete in vivo metabolism within 60 min into a more polar species. Although the structure of the main metabolite was not elucidated, it is possible that metabolic liability of **[**^**18**^**F]5** arose from the highly reactive acrylamide moiety either through a putative glutathione conjugation, or more generally, the formation of covalent bonds with cellular components (i.e. biomolecules) (Capuano and Fogliano 2011). The rapid and complete metabolic degradation of **[**^**18**^**F]5** negates the use of this compound for further in vivo evaluation. Due to metabolic instability, we did not elaborate the uptake of **[**^**18**^**F]5** in normal tissues with high glycogen storage including liver and skeletal muscle.

## Conclusions

We report the development of fluorine-containing analogue of CDg4 (**5** and **[**^**18**^**F]5**) and investigate its properties as a dual-modality fluorescence/PET imaging probe for glycogen in a panel of cancer cell lines. The fluorine-18 radiolabelled **[**^**18**^**F]5** was synthesised in an acceptable RCY n.d.c. (5.1 ± 0.9%), molar activity (A_m_: 7.6 GBq/μmol) and excellent RCP > 98%. Compound **5** accumulated in glycogen-containing cells visualised by fluorescence microscopy, and the signal was diminished by enzymatic digestion of carbohydrates using α-amylase; however, no correlation between the in vitro uptake of **[**^**18**^**F]5** and total glycogen level in a panel of cancer cell lines was observed. Evaluation of **[**^**18**^**F]5** in vivo showed the accumulation of radioactivity in the gallbladder and intestines and rapid metabolism in 60 min. The investigation of suitable radiolabelled molecules for binding glycogen, without the inclusion of a potent Michael acceptor moiety is ongoing.

## Methods

### General

Anhydrous solvents and reagents were purchased from Sigma Aldrich (Gillingham, UK) and were used without additional purification. N-methyl-N-(2-hydroxyethyl)-4-aminobenzaldehyde was purchased from abcr GmbH (Karlsruhe, Germany). Flash column chromatography purification was performed on silica gel (Merck Kieselgel 60 F_254_ 320–400 mesh). Thin Layer Chromatography (TLC) was performed on Merck aluminium-backed plates pre-coated with silica (0.2 mm, 60 F_254_) which were visualised by quenching of ultraviolet fluorescence (λ = 254 and 365 nm). ^1^H-NMR, ^13^C-NMR and ^19^F-NMR was obtained using a Bruker AV-400 spectrometer at a frequency of 400, 101 and 376 MHz, respectively. Chemical shifts (δ) are given in parts per million (ppm) and referenced to the appropriate residual solvent peaks. Signals are assigned as s, d, t, dt, m and br for singlet, doublet, triplet, double triplet, multiplet and broad respectively. Mass spectrometry was performed by the Mass Spectrometry Facility of the Chemistry Department of Imperial College London. Excitation (λ_ex_) and emission (λ_em_) spectra were obtained using an Infinite 200 PRO plate reader (Tecan, Männedorf, Switzerland).

### Chemical synthesis

#### 2-((4-Formylphenyl)(methyl)amino) ethyl 4-methylbenzenesulfonate (2)

To a solution of the *N*-methyl-*N*-(2-hydroxyethyl)-4-aminobenzaldehyde (567 mg, 3.16 mmol) and TEA (881 μL, 6.32 mmol) in dry DCM (10 mL) was added dropwise 4-toluenesulfonyl chloride (723 mg, 3.79 mmol) in DCM (5 mL). The reaction mixture was stirred under N_2_ at room temperature overnight, after which time it was concentrated in vacuo. The resulting material was purified by silica gel column chromatography (n-hexane: EtOAc, 1:1) to provide the product (780 mg, 74%) as a pale yellow solid. ^1^H NMR (400 MHz, Chloroform-*d*) δ 9.75 (s, 1H), 7.94–7.54 (m, 4H), 7.34–7.05 (m, 2H), 6.59 (d, *J* = 9.0 Hz, 2H), 4.21 (t, *J* = 5.7 Hz, 2H), 3.72 (t, *J* = 5.7 Hz, 2H), 3.01 (s, 3H), 2.39 (s, 3H). ^13^C NMR (101 MHz, Chloroform-*d*) δ 190.36, 152.76, 145.26, 132.62, 132.10, 129.99, 127.92, 126.12, 111.25, 66.57, 51.07, 39.45, 21.79. HRMS (ESI) = 334.1120 (M + H)^+^. Calc. for C_17_H_20_NO_4_S: 334.1113.

#### N-methyl-N-(2-fluoroethyl)-4-aminobenzaldehyde (3)

To a solution of 2-((4-formylphenyl)(methyl)amino)ethyl-4-methylbenzenesulfonate (760 mg, 2.28 mmol) in dry THF (15 mL) was added dropwise TBAF (3.6 mL, 1 M in THF, 3.6 mmol). The reaction mixture was stirred under N_2_ at 100 °C for 2 h, followed by cooling to room temperature and concentration in vacuo. The residue was taken up in DCM (20 mL), washed with NH_4_Cl (30 mL) and brine (30 mL), dried over Na_2_SO_4_. The organic layer was filtered and concentrated in vacuo. The residue was purified by silica gel column chromatography (100% DCM) to afford the product (400 mg, 97%) as a yellow solid. ^1^H NMR (400 MHz, Chloroform-*d*) δ 9.64 (s, 1H), 7.63 (d, *J* = 8.9 Hz, 2H), 6.63 (d, *J* = 8.9 Hz, 2H), 4.53 (dt, *J* = 47.1, 5.0 Hz, 2H), 3.65 (dt, *J* = 24.9, 5.0 Hz, 2H), 3.02 (s, 3H). ^13^C NMR (101 MHz, Chloroform-*d*) δ 190.25, 153.29, 132.03, 125.63, 111.15, 81.49 (d, *J* = 170.5 Hz), 52.16 (d, *J* = 20.8 Hz), 39.17. ^19^F NMR (376 MHz, Chloroform-*d*) δ − 222.55 (m). HRMS (ESI) = 182.0988 (M + H)^+^. Calc. for C_10_H_13_NO_4_F: 182.0981.

#### (E)-1-(4-Aminophenyl)-3-(4-((2-fluoroethyl)(methyl)amino)phenyl)prop-2-en-1-one (4)

To a stirred solution of N-Methyl-N-(2-fluoroethyl)-4-aminobenzaldehyde (386 mg, 2.13 mmol) and 4′-aminoacetophenone (262 mg, 1.94 mmol) in EtOH (20 mL) was added 4 M NaOH (0.5 mL) at RT. The reaction mixture was stirred at 50 °C for 16 h. The solution was then cooled and neutralised with cold 1 M HCl, followed by in vacuo removal of bulk solvent. The resulting material was purified by silica gel column chromatography (gradient n-hexane: EtOAc, 2:1, then 1:1) to provide the product (90 mg, 16%) as an orange solid. ^1^H NMR (400 MHz, Chloroform-*d*) δ 7.92 (d, *J* = 8.7 Hz, 2H), 7.75 (d, *J* = 15.4 Hz, 1H), 7.53 (d, *J* = 8.9 Hz, 2H), 7.36 (d, *J* = 15.4 Hz, 1H), 6.77–6.64 (m, 4H), 4.61 (dt, *J* = 47.1, 5.1 Hz, 2H), 3.70 (dt, *J* = 24.5, 5.2 Hz, 2H), 3.08 (s, 3H). ^13^C NMR (101 MHz, Chloroform-*d*) δ 188.43, 150.82, 150.43, 143.88, 130.91, 130.32, 129.29, 123.76, 117.38, 114.02, 111.98, 81.77 (d, *J* = 170.2 Hz), 52.44 (d, *J* = 21.0 Hz), 39.23. ^19^F NMR (376 MHz, Chloroform-*d*) δ − 222.25 (m). HRMS (ESI) = 299.1571 (M + H)^+^. Calc. for C_18_H_20_N_2_OF: 299.1560.

#### (E)-N-(4-(3-(4-((2-Fluoroethyl)(methyl)amino)phenyl)acryloyl)phenyl) acrylamide (5)

To a mixture of compound **4** (80 mg, 0.27 mmol) and TEA (90 μL, 0.64 mmol) in acetonitrile (2 mL) was added the acryloyl chloride (50 μL, 0.64 mmol) at RT and stirred for 2 h. After concentration in vacuo, the residue was purified by silica gel chromatography (1% MeOH in DCM) to provide the product (10 mg, 11%) as a yellow solid. ^1^H NMR (400 MHz, Chloroform-*d*) δ 8.02 (d, *J* = 8.8 Hz, 2H), 7.78 (d, *J* = 15.5 Hz, 1H), 7.73 (d, *J* = 8.7 Hz, 2H), 7.55 (d, *J* = 8.9 Hz, 2H), 7.35 (d, *J* = 15.4 Hz, 1H), 6.70 (d, *J* = 8.9 Hz, 2H), 6.48 (dd, *J* = 16.9, 1.3 Hz, 1H), 6.30 (dd, *J* = 16.9, 10.2 Hz, 1H), 5.82 (dd, *J* = 10.2, 1.3 Hz, 1H), 4.63 (dt, *J* = 47.1, 5.1 Hz, 2H), 3.72 (dt, *J* = 24.5, 5.1 Hz, 2H), 3.10 (s, 3H). ^13^C NMR (101 MHz, Chloroform-*d*) δ 189.35, 163.80, 150.81, 145.56, 141.64, 134.90, 130.99, 130.69, 129.88, 128.82, 123.37, 119.36, 117.07, 112.02, 81.76 (d, *J* = 170.4 Hz), 52.46 (d, *J* = 21.2 Hz), 39.30. ^19^F NMR (376 MHz, Chloroform-*d*) δ − 222.31 (m). HRMS (ESI) = 353.1659 (M + H)^+^. Calc. for C_21_H_22_N_2_O_2_F: 353.1665.

#### Tert-butyl (4-acetylphenyl) carbamate (7)

To a solution of the 4′-aminoacetophenone (1.5 g, 11.1 mmol) and TEA (5.6 mL, 39.96 mmol) in dry DCM (20 mL) was added di-tert-butyl dicarbonate (2.7 g, 12.21 mmol) in DCM (5 mL). The reaction mixture was stirred under N_2_ at RT for 16 h, after which time it was concentrated in vacuo. The resulting material was purified by silica gel column chromatography (DCM:EtOAc, 20:1) to provide the product (1.58 g, 61%) as a pale white solid. 1H NMR (400 MHz, Chloroform-d) δ 7.91 (d, J = 8.8 Hz, 2H), 7.45 (d, J = 8.8 Hz, 2H), 6.70 (s, 1H), 2.56 (s, 3H), 1.53 (s, 9H). 13C NMR (101 MHz, Chloroform-d) δ 197.00, 152.27, 143.02, 132.02, 129.99, 117.54, 81.46, 28.42, 26.52. HRMS (ESI) = 236.1287 (M + H)+. Calc. for C_13_H_18_NO_3_: 236.1287.

#### tert-Butyl (E)-(4-(3-(4-((2-hydroxyethyl)(methyl)amino)phenyl)acryloyl)phenyl) carbamate (8)

To a stirred solution of compound 7 (250 mg, 1.07 mmol) was added NaOH (10 mg) and *N*-methyl-*N*-(2-hydroxyethyl)-4-aminobenzaldehyde (287 mg, 1.61 mmol) in EtOH (5 mL) at RT. The reaction mixture was stirred at 50 °C for 16 h, followed by in vacuo removal of bulk solvent. The resulting material was purified by silica gel column chromatography (gradient DCM: EtOAc, 20:1, then 1:1) to provide the product (131 mg, 30%) as a pale yellow solid. ^1^H NMR (400 MHz, Chloroform-*d*) δ 7.97 (d, *J* = 8.7 Hz, 2H), 7.74 (d, *J* = 15.5 Hz, 1H), 7.49 (m, 4H), 7.31 (d, *J* = 15.4 Hz, 1H), 6.88 (s, 1H), 6.73 (d, *J* = 8.9 Hz, 2H), 3.84 (t, *J* = 5.7 Hz, 2H), 3.55 (t, *J* = 5.8 Hz, 2H), 3.04 (s, 3H), 1.53 (s, 9H). ^13^C NMR (101 MHz, Chloroform-*d*) δ 189.25, 152.43, 151.47, 145.29, 142.47, 133.48, 130.57, 129.94, 123.23, 117.66, 116.88, 112.14, 81.28, 60.24, 54.70, 39.08, 28.41. HRMS (ESI) = 397.2113 (M + H)^+^. Calc. for C_23_H_29_N_2_O_4_: 397.2127.

#### (E)-2-((4-(3-(4-((tert-Butoxycarbonyl)amino)phenyl)-3-oxoprop-1-en-1-yl)phenyl)(methyl)amino) ethyl 4-methylbenzenesulfonate (9)

Compound **8** (115 mg, 0.29 mmol) was dissolved in anhydrous pyridine (8 mL) under N_2_. The solution was stirred at 0 °C and 4-toluenesulfonyl chloride (110.6 mg, 0.58 mmol) in pyridine (1 mL) was added slowly. Reaction was stirred for 4 h at 0 °C and then at RT for 16 h. After completion of the reaction, the mixture was partitioned between EtOAc (15 mL) and H_2_O (15 mL). The organic layer was washed with H_2_O (2 × 10 mL), 1 M HCl solution (2 × 10 mL), brine (2 × 10 mL) and dried over Na_2_SO_4_. The solvent was removed in vacuo. The resulting residue was purified by silica gel column chromatography (n-hexane: EtOAc, 1:3) to provide the product (100 mg, 63%) as a pale yellow solid. ^1^H NMR (400 MHz, Chloroform-*d*) δ 7.98 (d, *J* = 8.8 Hz, 2H), 7.73 (d, *J* = 15.5 Hz, 1H), 7.66 (d, *J* = 8.4 Hz, 2H), 7.51 (d, *J* = 8.8 Hz, 2H), 7.44 (d, *J* = 8.9 Hz, 2H), 7.33 (d, *J* = 15.4 Hz, 1H), 7.25–7.17 (m, 2H), 7.13 (s, 1H), 6.52 (d, *J* = 8.9 Hz, 2H), 4.16 (t, *J* = 5.7 Hz, 2H), 3.63 (t, *J* = 5.8 Hz, 2H), 2.91 (s, 3H), 2.35 (s, 3H), 1.50 (s, 9H). ^13^C NMR (101 MHz, Chloroform-*d*) δ 188.95, 152.44, 149.97, 145.07, 144.82, 142.66, 133.22, 132.51, 130.35, 129.86, 129.83, 127.79, 123.46, 117.61, 117.14, 111.79, 81.05, 66.80, 50.91, 39.09, 28.33, 21.67. HRMS (ESI) = 551.2218 (M + H)^+^. Calc. for C_30_H_35_N_2_O_6_S: 551.2210.

### Fluorescence

Compound **5** was freshly prepared in DMSO at the indicated concentrations of 0.01, 0.1, 1, 10 and 50 μM. Each concentration was assayed in triplicate in a 96-well dark plate using a plate reader (Infinite 200 PRO, Tecan). Absorption spectra were first determined, where the absorbance was plotted against the measured wavelength ranging from 380 nm to 460 nm. The wavelength of maximum absorption was used as the excitation wavelength to obtain emission (fluorescence) spectra of compound **5**. All spectra shown in ESI.

### Radiosynthesis of [^18^F]5

The radiochemistry for the synthesis of **[**^**18**^**F]5** was partly automated using the GE FASTLab™ platform. An automated sequence was developed (ESI, Figure S19) to trap [^18^F] fluoride in oxygen-18 water on a QMA-bicarbonate SPE cartridge, which was eluted into the reactor vessel using a 4:1 solution (1 mL) of K_222_ (12 mg/mL) and KHCO_3_ (18 mg/mL) respectively. The [^18^F] fluoride was dried by evaporation at 120 °C under vacuum and a flow of nitrogen for 8 min then 70 °C for 5 min. To the dry fluoride was added precursor **9** (1.6 mg) in MeCN (1.2 mL) and the reactor sealed and heated to 90 °C for 20 min, after which the reactor was cooled. The crude reaction mixture containing intermediate **[**^**18**^**F]10** was expelled into an external dilution vial containing H_2_O (25 mL) and loaded through a preconditioned tC18 SPE cartridge [conditioned with 5 mL MeCN and 10 mL H_2_O prior to the experiment]. The cartridge was subsequently washed with H_2_O (10 mL), dried under nitrogen for 60 s, and the radioactive product eluted into a Wheaton vial with MeCN (1 mL) and analysed by analytical radio-HPLC (Figure S20). The following radiochemistry was performed by hand. To the Wheaton vial containing **[**^**18**^**F]10** was added phosphoric acid (1 mL, 1.8 M) and heated to 80 °C for 15 min. Hydrolysis of the boc-protecting group to yield **[**^**18**^**F]4** was confirmed by analytical radio-HPLC analysis (Figure S21). The reaction mixture containing **[**^**18**^**F]4** was diluted in H_2_O (25 mL) and trapped on a preconditioned tC18 SPE cartridge [conditioned with 5 mL MeCN and 10 mL H_2_O prior to the experiment]. The cartridge was washed with H_2_O (10 mL) and dried under the vigorous flow of nitrogen for 5 mins. Compound **[**^**18**^**F]4** was eluted into a dry Wheaton vial using anhydrous MeCN (1 mL) followed by the addition of acryloyl chloride (50 μL), the vessel was heated to 50 °C for 15 min and reaction progress monitored by analytical radio-HPLC (Figure S22). Once complete, the crude reaction mixture was diluted in H_2_O containing 0.1% TFA (9 mL) and purified by semi-preparative HPLC (mobile phase: 60% MeCN/ 40% H_2_O / 0.1% TFA) with the desired product eluting at t_R_ = 11 min. The desired product was cut into H_2_O (30 mL) and trapped on a preconditioned tC18 SPE [conditioned with 5 mL EtOH and 10 mL H_2_O prior to the experiment]. The final product (**[**^**18**^**F]5**) was eluted in EtOH (0.5–1 mL) into a clean vial for biological evaluation. Identity of **[**^**18**^**F]5** was confirmed by radio-HPLC analysis and co-elution with the authentic reference compound **5** (Figure S23).

### Radioactive metabolite analysis

Liver, urine and blood plasma were analysed for radioactive metabolites by radio-HPLC (Agilent 1100 system) fitted with an in-line posiRAM metabolite detector (Lablogic, Sheffield, UK). An isocratic mobile phase (60% MeCN / 40% H_2_O / 0.1% TFA, 3 mL/min) was used in conjunction with an Agilent Zorbax XDB C18 column (250 × 9.4 mm, 5 μ). The retention time of parent compound **[**^**18**^**F]5** was determined by injecting a radioactive sample onto the metabolite radio-HPLC system. The liver was excised, and a portion homogenised in ice cold MeCN (1 mL) using a Precellys tissue homogeniser fitted with the Cryolys cooling module (Stretton Scientific Ltd., Derbyshire, UK). Solid tissues and protein were pelleted by centrifugation (13,000 g, 5 min) and the supernatant was removed and filtered (0.22 μm syringe filter) before being diluted in H_2_O + 0.1% TFA for radio-HPLC analysis. Urine was diluted in H_2_O + 0.1% TFA and filtered prior to radio-HPLC analysis. Plasma was obtained from whole blood by centrifugation (2000 g, 5 min) to separate the blood cells from the plasma; the plasma was removed and precipitated in ice cold MeCN (1 mL) and centrifuged (13,000 g, 5 min) to pellet the proteins. The supernatant was filtered (0.22 μm syringe filter) and diluted in H_2_O + 0.1% TFA for radio-HPLC analysis. The HPLC injection loop (1 mL) was washed with mobile phase between each injection. The efficiency of extracting radioactivity from each plasma and liver sample was determined by counting the activity (counts per minute, CPM) in a small aliquot (20 μL) of the supernatant of a known volume and the whole protein pellet, in a λ-counter (PerkinElmer, Wizard2). The extraction efficiency of radioactivity from plasma and liver was 73.7 ± 2.5 and 45.2 ± 4.3% of total activity, respectively. Radio-HPLC chromatograms were integrated using Laura 6 software (Lablogic, Sheffield, UK).

### Cell culture

SKOV-3 (ovarian carcinoma; ATCC), MCF-7 (breast carcinoma; ATCC), T47D (breast carcinoma; ATCC), MDA-MB-231 (breast carcinoma; ATCC), HCT116 (colorectal carcinoma; ATCC), 786-O (clear cell renal carcinoma; ATCC), RCC4 plus vector alone (−VHL, clear cell renal carcinoma; ECACC), RCC4 plus VHL (+VHL, clear cell renal carcinoma; ECACC), U87 shCtrl and U87 shPYGL (glioblastoma; kind gifts of Prof. Adrian Harris, University of Oxford) were cultured in DMEM media (Sigma-Aldrich) supplemented with 10% foetal calf serum (FCS), 1% L-glutamine and 2% penicillin-streptomycin. IGROV-1 (ovarian carcinoma; ATCC) was maintained in RPMI 1640 media (Sigma-Aldrich) with the same supplements as described above. All cell lines were cultured at 37 °C in a humidified atmosphere containing 5% CO_2_. All cell lines were routinely tested for mycoplasma and typically not passaged for longer than 3 months.

### Quantification of total glycogen

Cells were each seeded at an appropriate density and allowed to attach overnight. After 24 h, cells were trypsinised, centrifuged and washed to obtain pellets. Glycogen was extracted by boiling cell pellets in 30% KOH for 15 min. The mixture, in the presence of 2% Na_2_SO_4_ and absolute ethanol, was kept in fridge overnight to induce glycogen precipitation. On the next day, the mixture was centrifuged and re-incubated with 70% ethanol for 30 min to purify the precipitation. After the last centrifuge, glycogen precipitates were dissolved in deionised water and ready for glycogen assay. Amyloglucosidase was firstly added to break down glycogen into glucose. Then the resulted glucose was measured using a Glucose (GO) assay kit (Sigma) according to manufacturer’s instructions. Assay reagent was added to samples and incubated at 37 °C for 45 min. 12 N sulfuric acid (H_2_SO_4_) was added to terminate the reaction and absorbance was measured at 540 nm using a standard microplate reader. Glycogen levels in unknown samples were determined based on the glycogen standard curve and normalised to total intracellular protein as measured by BCA.

### Fluorescence staining

At 24 h after seeding, fresh media containing 4 μM or 8 μM of compound **5** was added to cells and incubated at 37 °C for 1 h in dark. Hoechst 33342 (2 μg/mL) was used as nuclear staining. After 1 h incubation, cells were gently washed twice with PBS and images were obtained using a standard fluorescence microscope (Olympus BX51). For glycogen digestion, fresh media containing 0.5 mg/mL of α-amylase was added to cells after the 1 h-staining. Control cells were incubated in normal media for 15 min, in the absence of α-amylase.

### In vitro uptake assay

Cells were seeded at each appropriate density and allowed to attach for 24 h. On the day of uptake, cells were washed three times with pre-warmed PBS and incubated with 1 mL of fresh DMEM media containing approximately 0.74 MBq **[**^**18**^**F]5** for 1 h at 37 °C in a humidified condition of 5% CO_2_. Cells were then washed three times with ice-cold PBS and lysed in 1 mL of RIPA buffer for 10 min on ice. The radioactivity of 800 μL lysate from each sample was counted on a WIZARD2 Automatic Gamma Counter. Data were expressed as a percentage of radioactivity incorporated into cells, normalised to total cellular protein as measured by BCA assay.

### In vivo PET imaging

For PET imaging, BALB/c mice (Charles River UK Ltd., Margate, UK) were anesthetized and scanned on a dedicated small animal PET scanner (G4 Genesis, Sofie Biosciences, Culver City, CA, USA) following a bolus injection of 1.48 MBq of **[**^**18**^**F]5** via a lateral tail vein cannula. Imaging was performed under 2% isoflurane/O2 anaesthesia. After tracer injection, emission scans were acquired in list-mode format (over 0–60 min - dynamic scans) to give decay-corrected values of radioactivity accumulation in tissues. The collected data were reconstructed with a 3-dimensional maximum likelihood estimation method 3D ML-EM (Sofie Biosciences). Cumulative images of the data were used for visualization of radiotracer uptake and to define tissue volumes of interest (VOIs) using Siemens Inveon Research Workplace software (Siemens Molecular Imaging, Inc. Knoxville, USA). Tissue radioactivity uptake values were normalized to injected dose and mouse weight.

### Statistical analysis

Data were expressed as mean values ± standard deviation (SD). Correlation analysis was determined using GraphPad Prism v.6.

## Supplementary information

**Additional file 1 **Materials and methods; ^1^H, ^13^C and ^19^F NMR data; UV-Vis spectra; detailed radiochemistry and automation methods for the synthesis of **[**^**18**^**F]5**; UV-HPLC and radio-HPLC chromatograms.

## Data Availability

All data generated or analysed in this study are included in this published article or the associated supplementary information file.

## Abbreviations

A_m_: Molar activity
BCA: Bicinchoninic acid assay
DCM: Dichloromethane
EtOAc: Ethyl acetate
FACS: Fluorescence assisted cell sorting
TBAF: *Tert*-butylammonium fluoride
PGP: Permeability glycoprotein
RCP: Radiochemical purity
RCY: Radiochemical yield, non-decay corrected to start of radiosynthesis
RIPA: Radioimmunoprecipitation assay buffer
r.t.: Room temperature
SD: Standard deviation
TEA: Triethylamine
TFA: Trifluoroacetic acid
