# Supplementary material for: Development of a fluorine-18 radiolabelled fluorescent chalcone: evaluated for detecting glycogen
Source: EJNMMI Radiopharm Chem. 2020 Jun 23;5:17. doi: 10.1186/s41181-020-00098-6 (PMC7311592; doi:10.1186/s41181-020-00098-6)
Supplement: Supplementary file 1 — Additional file 1 Materials and methods; 1H, 13C and 19F NMR data; UV-Vis spectra; detailed radiochemistry and automation methods for the synthesis of [18F]5; UV-HPLC and radio-HPLC chromatograms. [file 41181_2020_98_MOESM1_ESM.docx]

Supporting Information

**Development of a fluorine-18 radiolabelled fluorescent chalcone:
 evaluated for detecting glycogen**

Louis Allott^Ϯ1^, Diana Brickute^Ϯ1^, Cen Chen^1^, Marta Braga^1^, Chris Barnes^1^, Ning Wang^1^,
 and Eric O. Aboagye^1^*

1. Comprehensive Cancer Imaging Centre, Imperial College London, Hammersmith Hospital, Du Cane Road, London, UK

*Corresponding Author: eric.aboaogye@imperial.ac.uk

^Ϯ^ Equal contribution

**Contents**

1.0 Materials and methods……………………………………………………………………………………………………..Page 02

2.0 NMR Spectra……………………………………………………………………………………………………………………..Page 03

3.0 UV-Vis/fluorescence Spectroscopy.…………………………………………………………………………………..Page 10

4.0 Radiochemistry………………………………………..……………………………………………………..………………..Page 11

5.0 HPLC Chromatograms……………………………………………………………………………………………………….Page 12

1. **Materials and methods**

^1^H, ^13^C and ^19^F NMR spectra were obtained using a Bruker 400 MHz spectrometer operating at room temperature. Chemical shifts (δ) are reported in parts per million (ppm) and residual solvent peaks have been used as an internal reference. Peak multiplicities have been abbreviated as follows: s (singlet), d (doublet), dd (double-doublet), m (multiplet). Excitation (λ_ex_) and emission (λ_em_) spectra were obtained using an Infinite 200 PRO plate reader (Tecan, Männedorf, Switzerland). NMR spectra were analysed using MestReNova v11 (Santiago de Compostela, Spain). Reaction efficiency and radioactive product identity was determined by RP-HPLC using an Agilent 1200 series instrument connected to a flow-ram detector (Lablogic, Sheffield, UK). The system was equipped with a Phenomenex Gemini 5μ C18 110 Å (150 × 4.6 mm) column; the mobile phase was A: H_2_O (0.1% TFA) and B: MeCN. The gradient was: 0 – 1 min, 95% A. 2– 10 min, 5% A. 11 – 20 min, 5% A at 1 mL/min. Elution profiles were analysed using Laura software (Lablogic, Sheffield, UK). Semi-preparative RP-HPLC was performed using a Shumadzu LC20-AT pump attached to a custom-built system, equipped with an Agilent Eclipse XDB-C18, 5μ (250 x 9.4 mm) column. The mobile phase was 60 % MeCN / 40 % H_2_O / 0.1% TFA (v/v), at 3 mL/min.

1. **NMR Spectra**


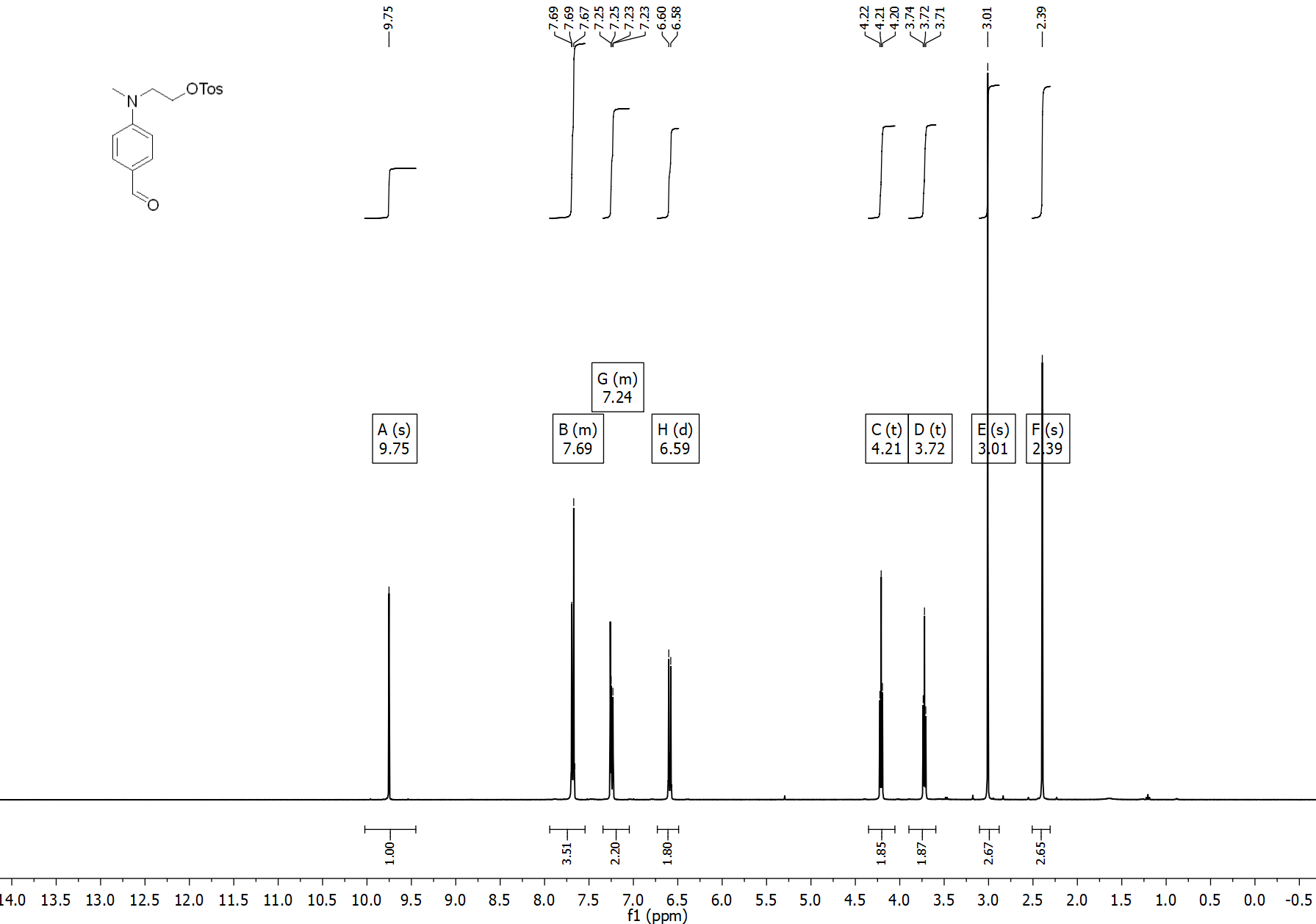


**Figure 1.** ^1^H-NMR spectra of compound **2**.


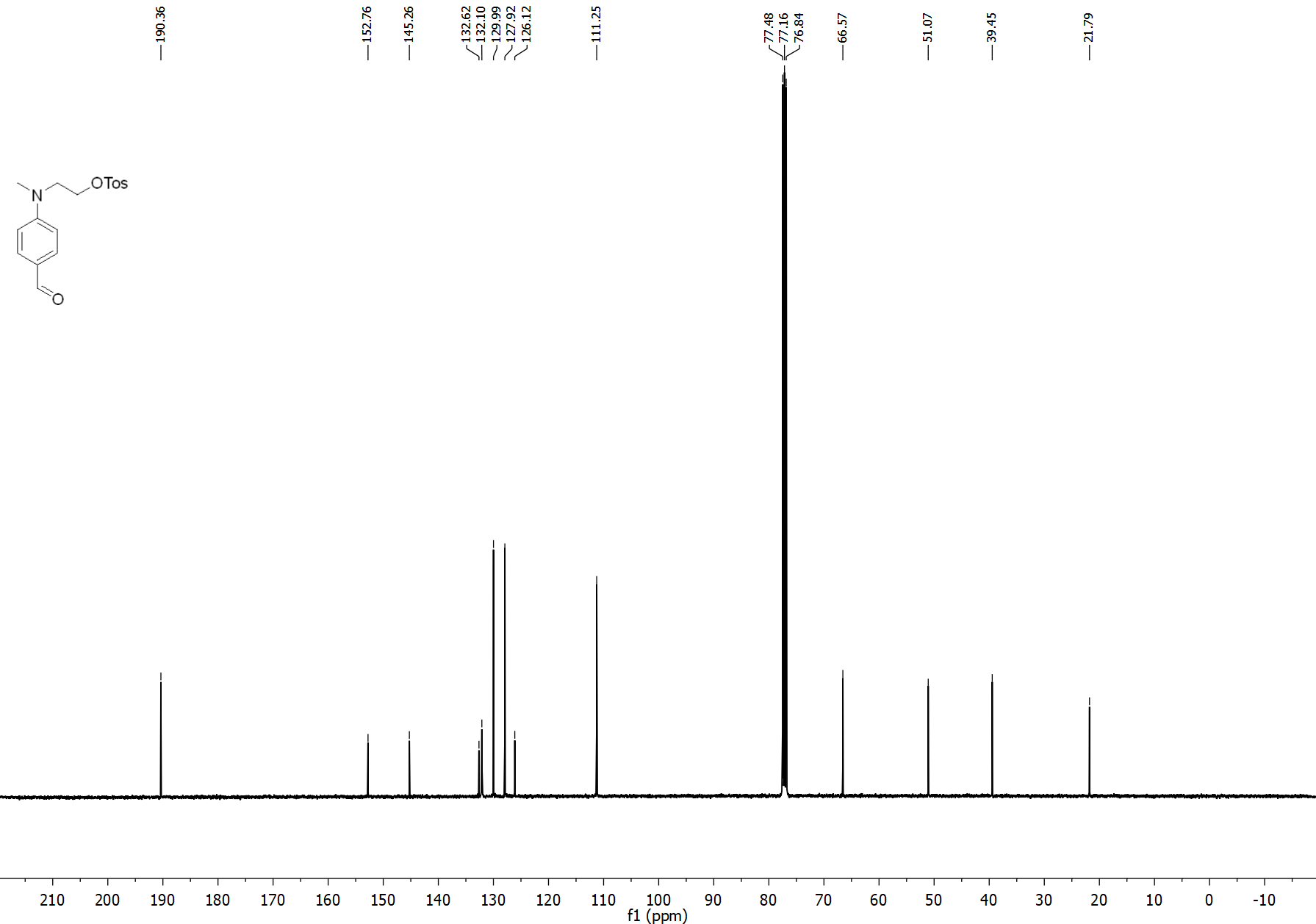


**Figure 2.** ^13^C-NMR spectra of compound **2**.


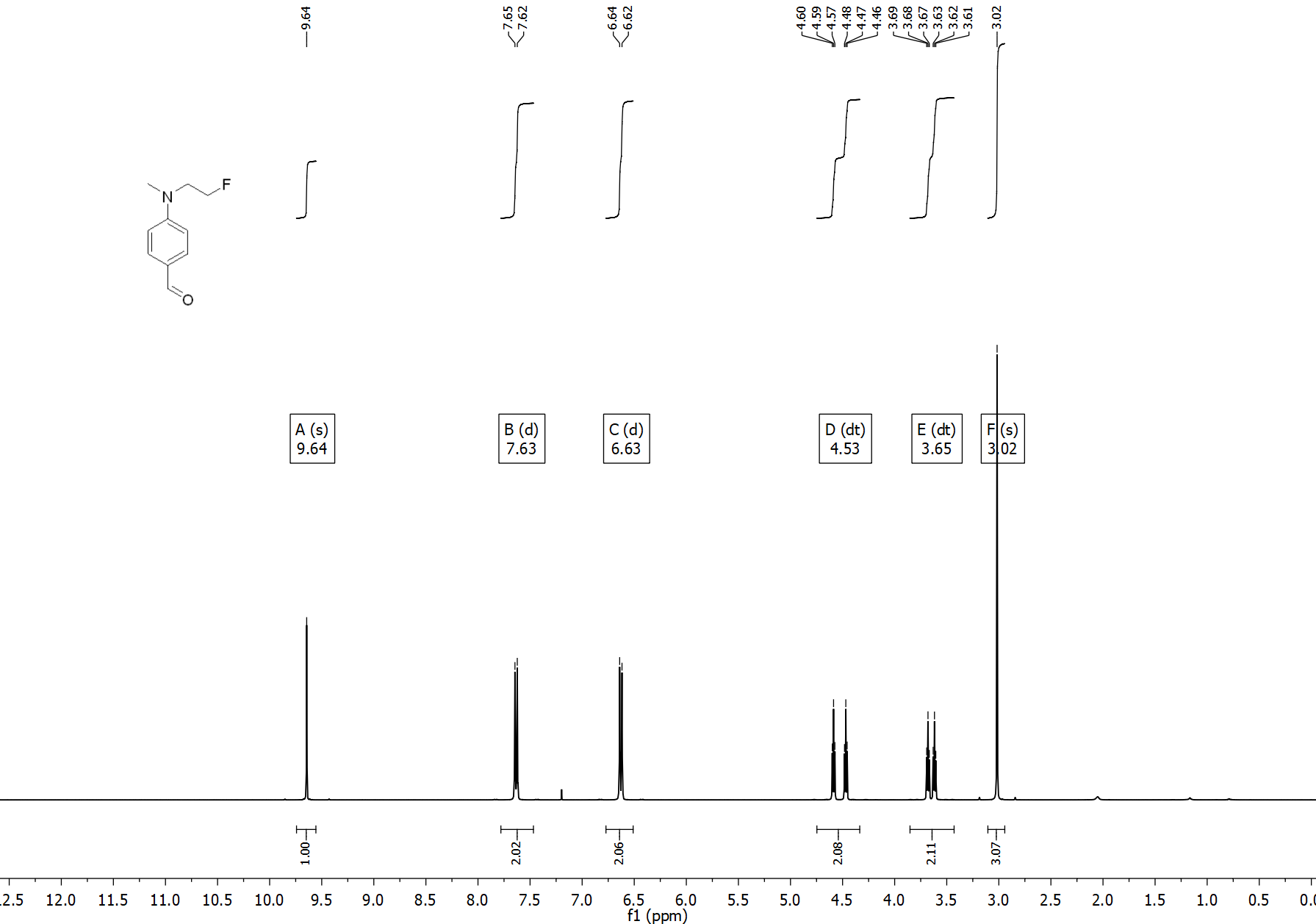


**Figure 3.** ^1^H-NMR spectra of compound **3**.


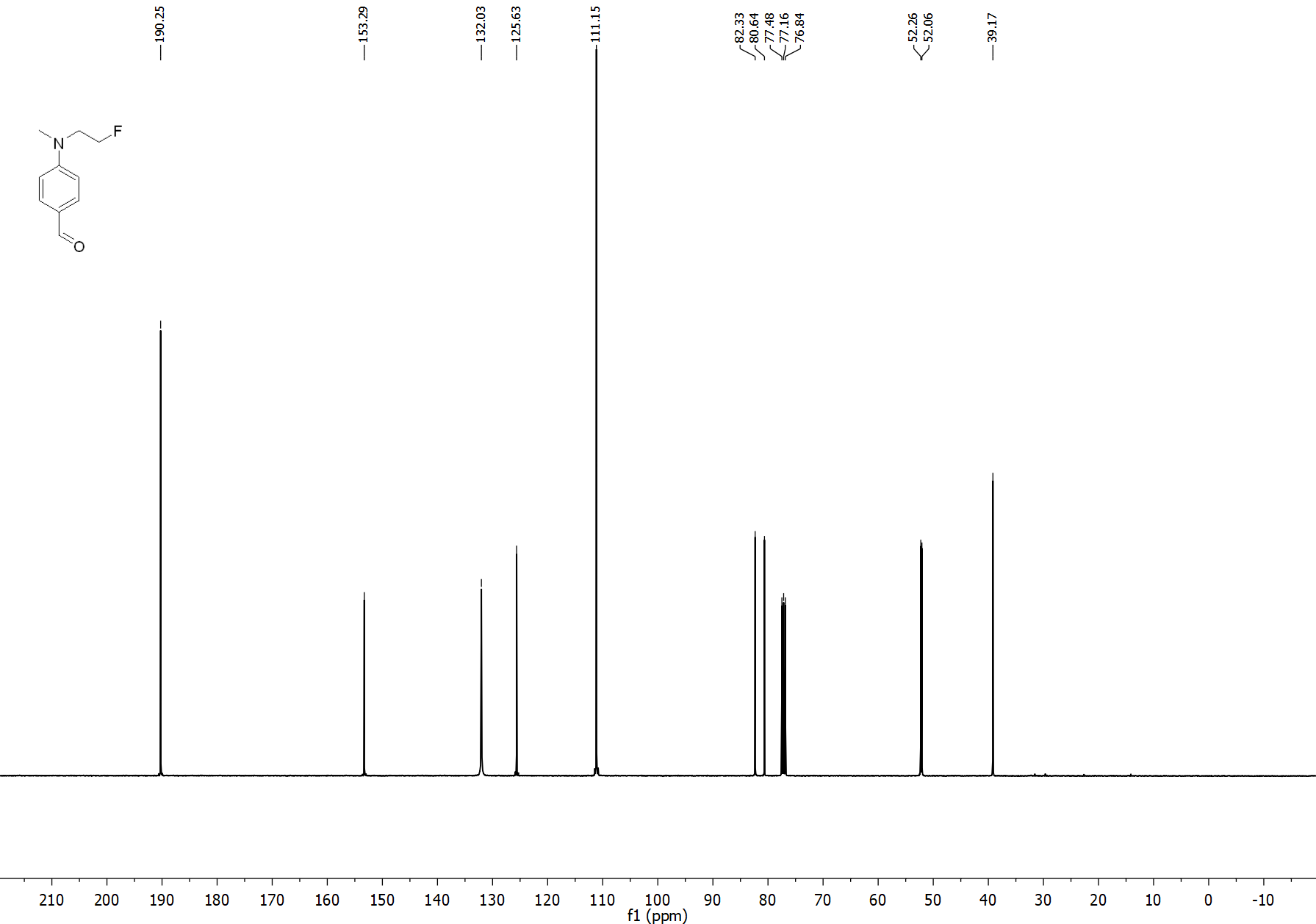


**Figure 4.** ^13^C-NMR spectra of compound **3**.


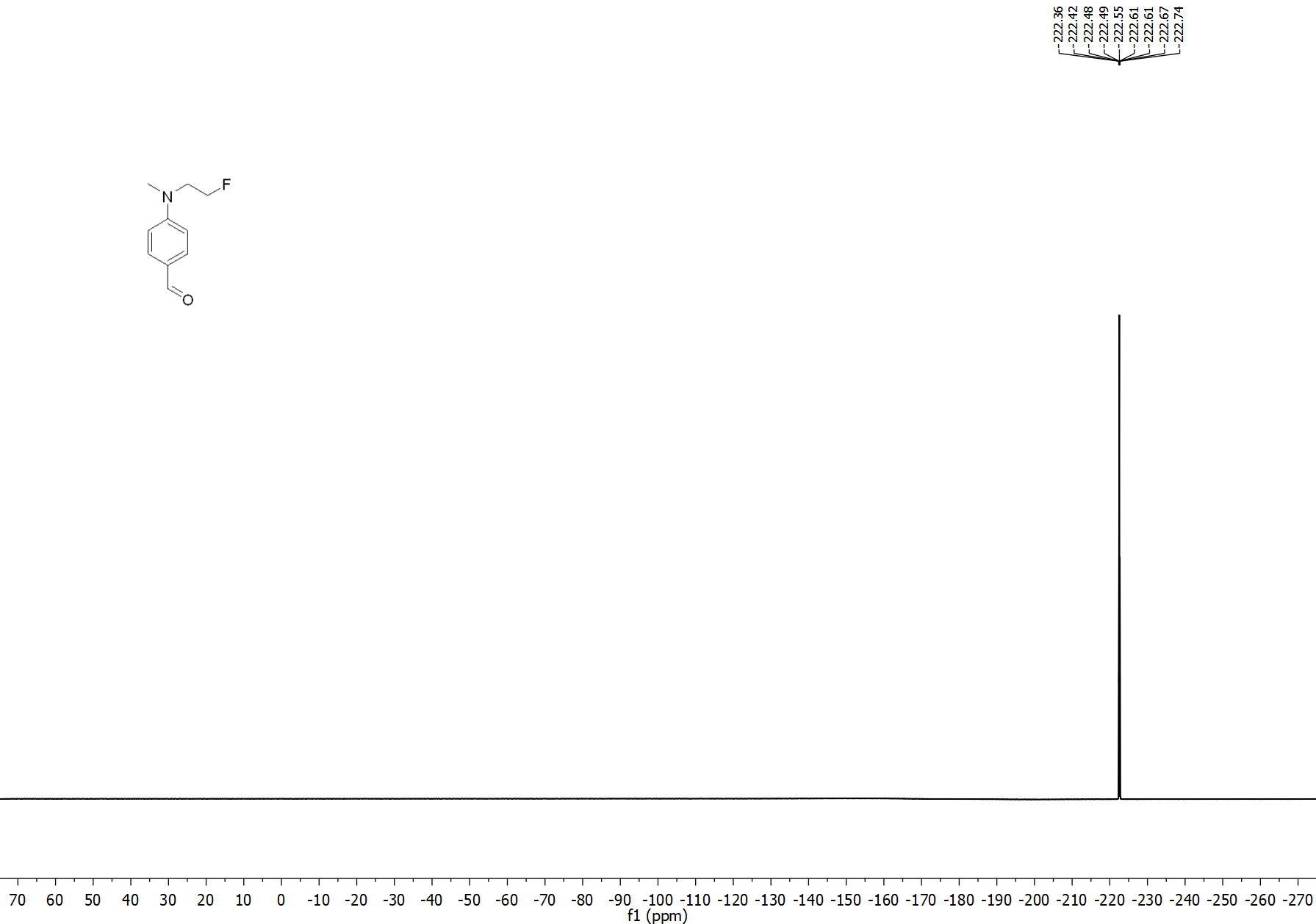
**Figure 5.** 19F-NMR spectra of compound **3**.


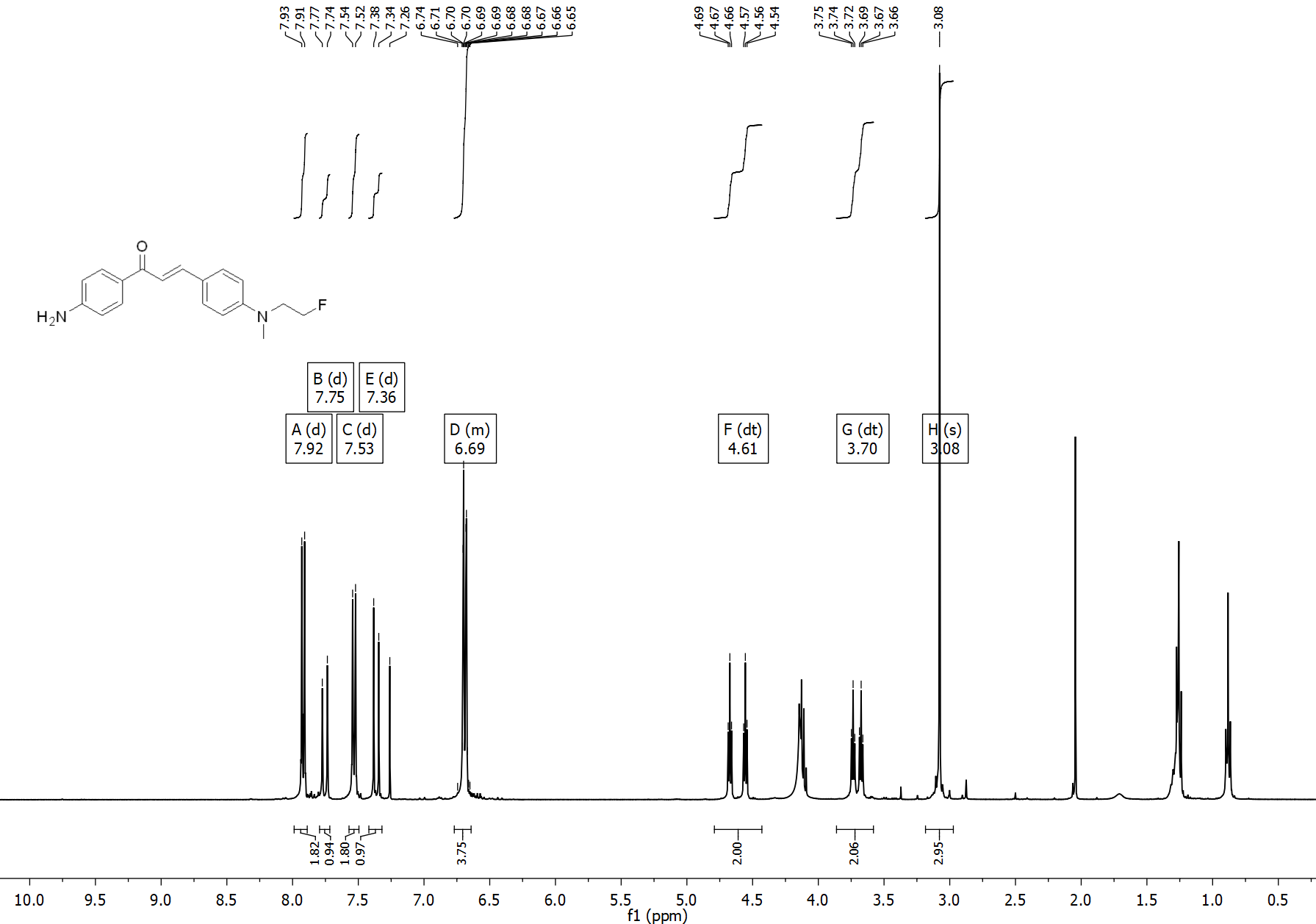


**Figure 6.** ^1^H-NMR spectra of compound **4**.


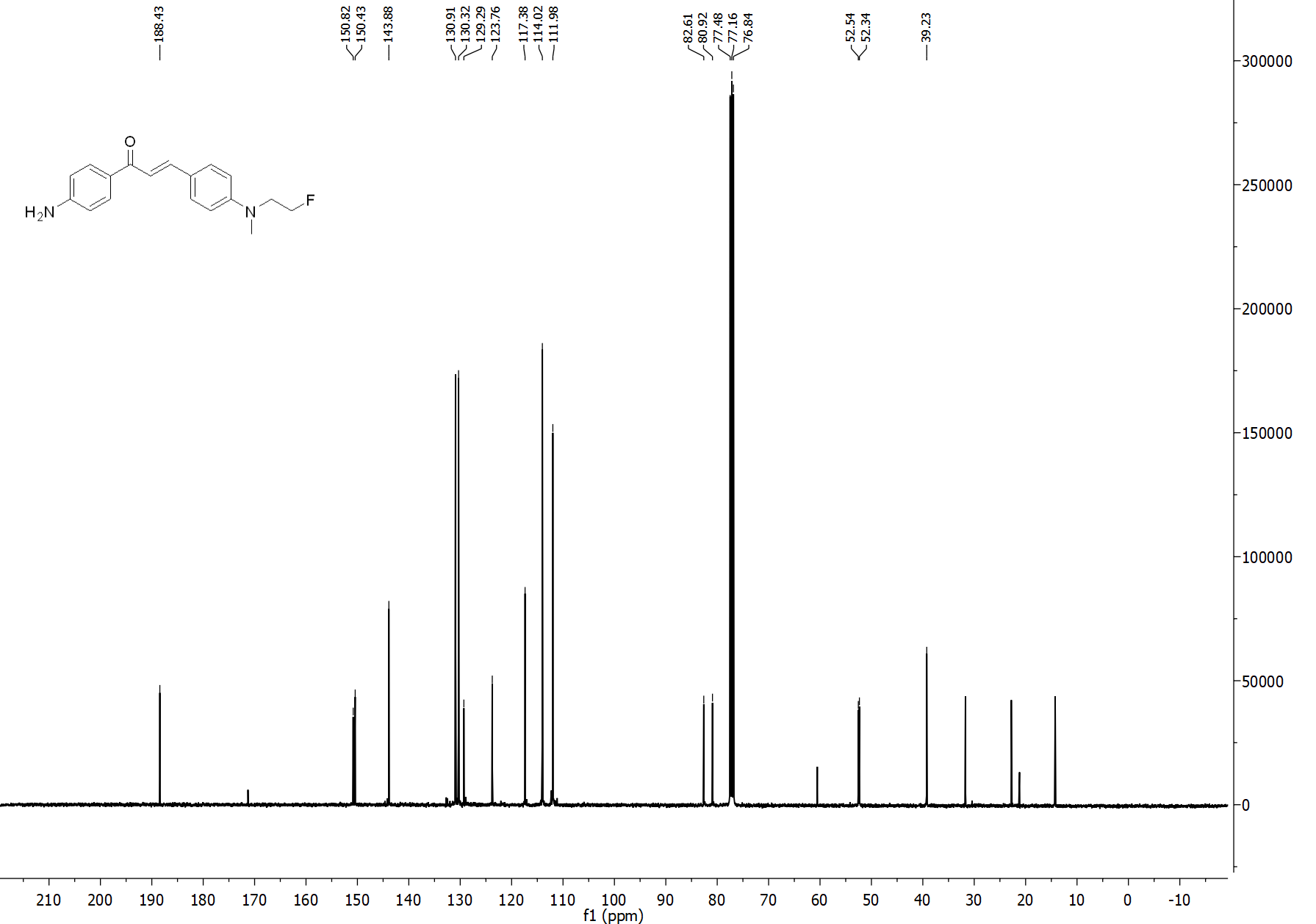


**Figure 7.** ^13^C-NMR spectra of compound **4**.


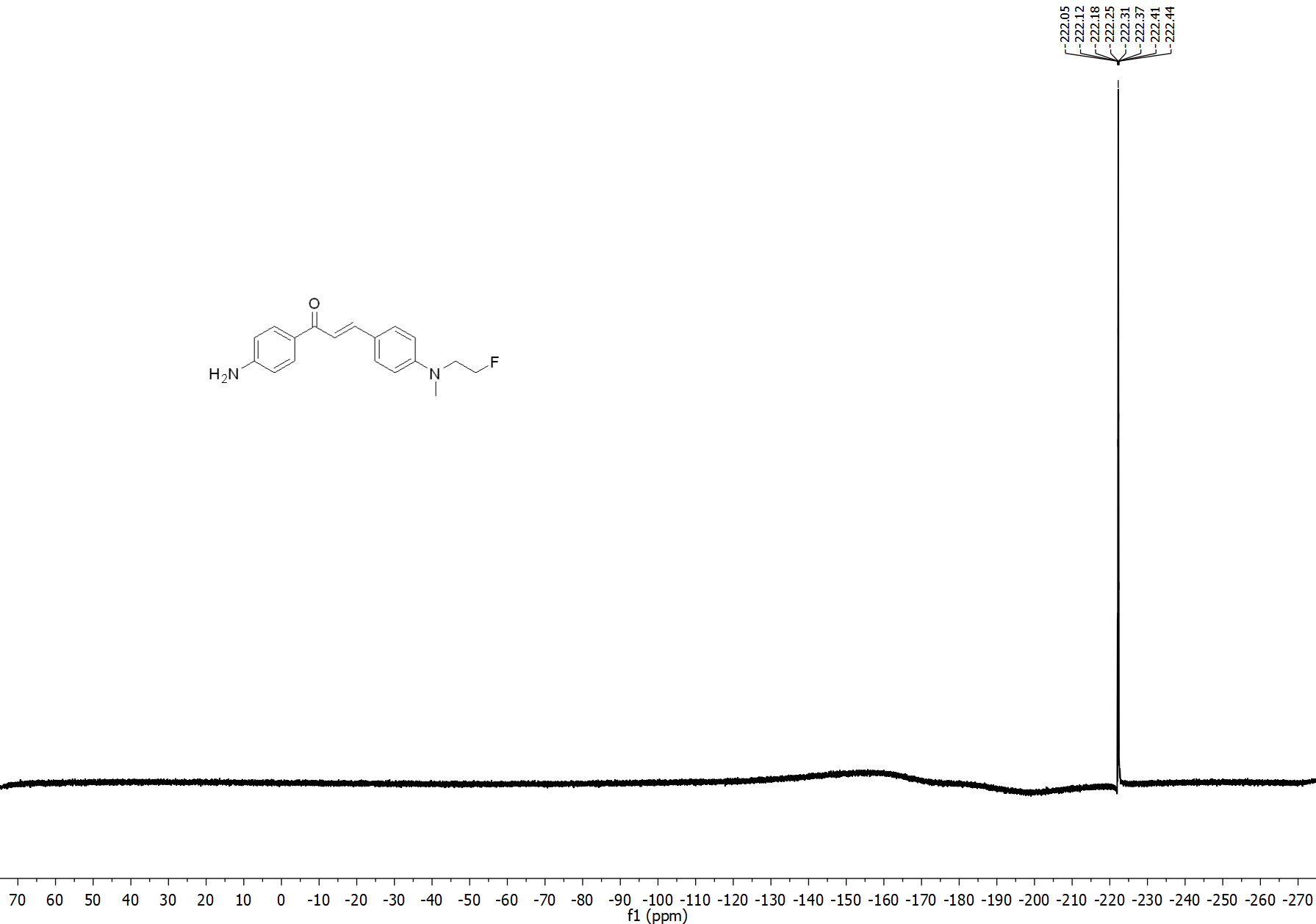


**Figure 8.** ^19^F-NMR spectra of compound **4**.


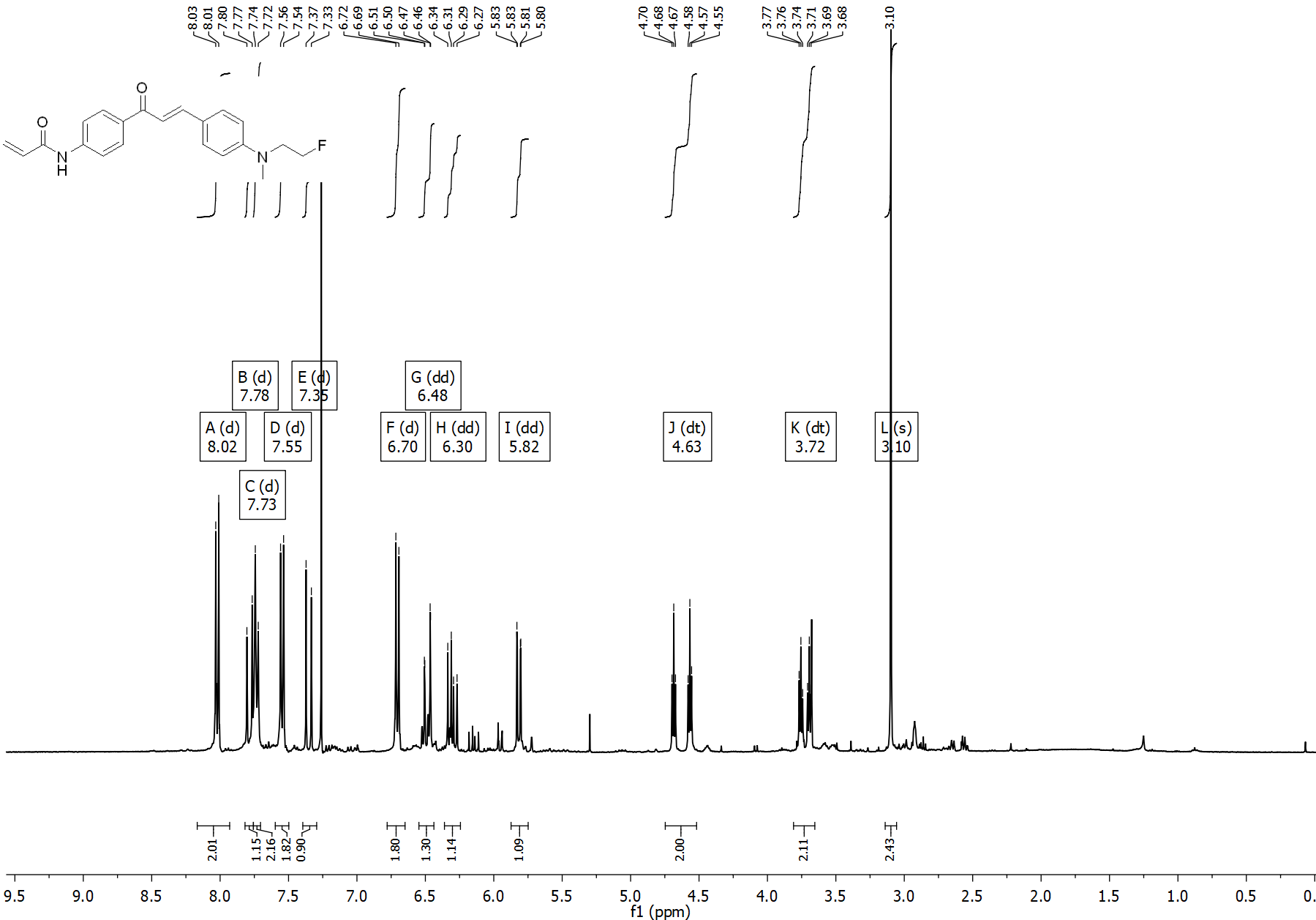


**Figure 9.** ^1^H-NMR spectra of compound **5**.


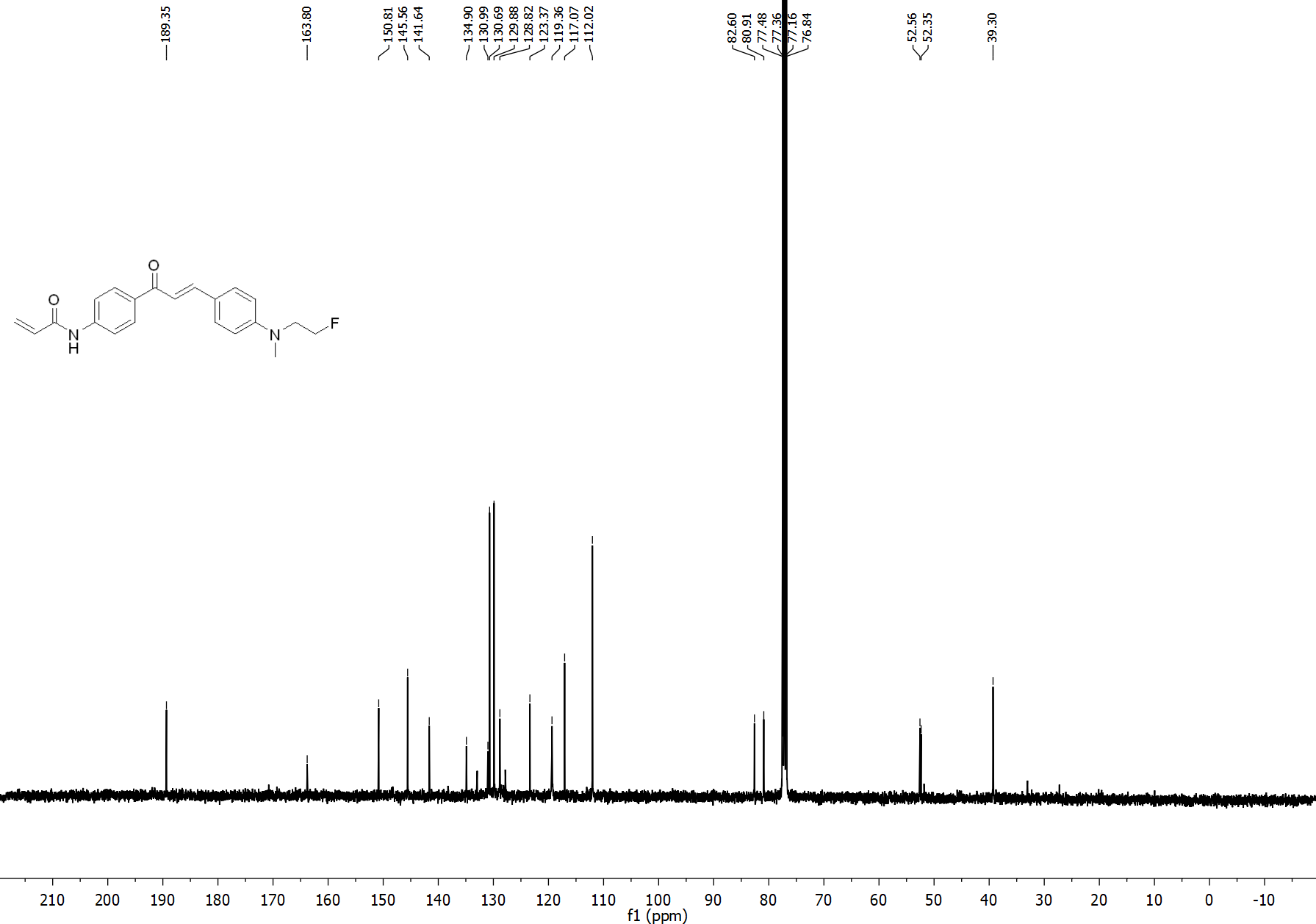


**Figure 10.** ^13^C-NMR spectra of compound **5**.


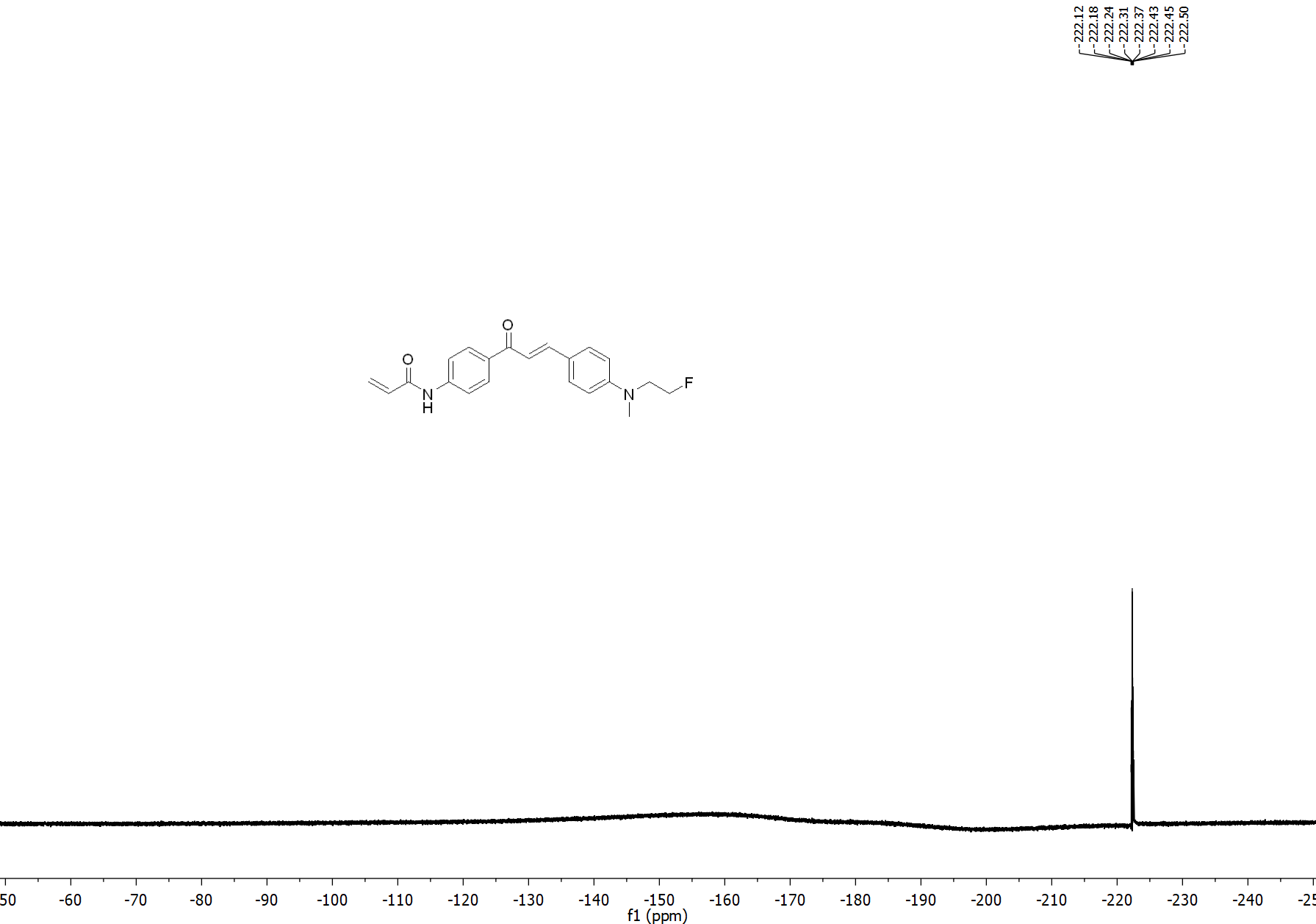


**Figure 11.** ^19^F-NMR spectra of compound **5**.


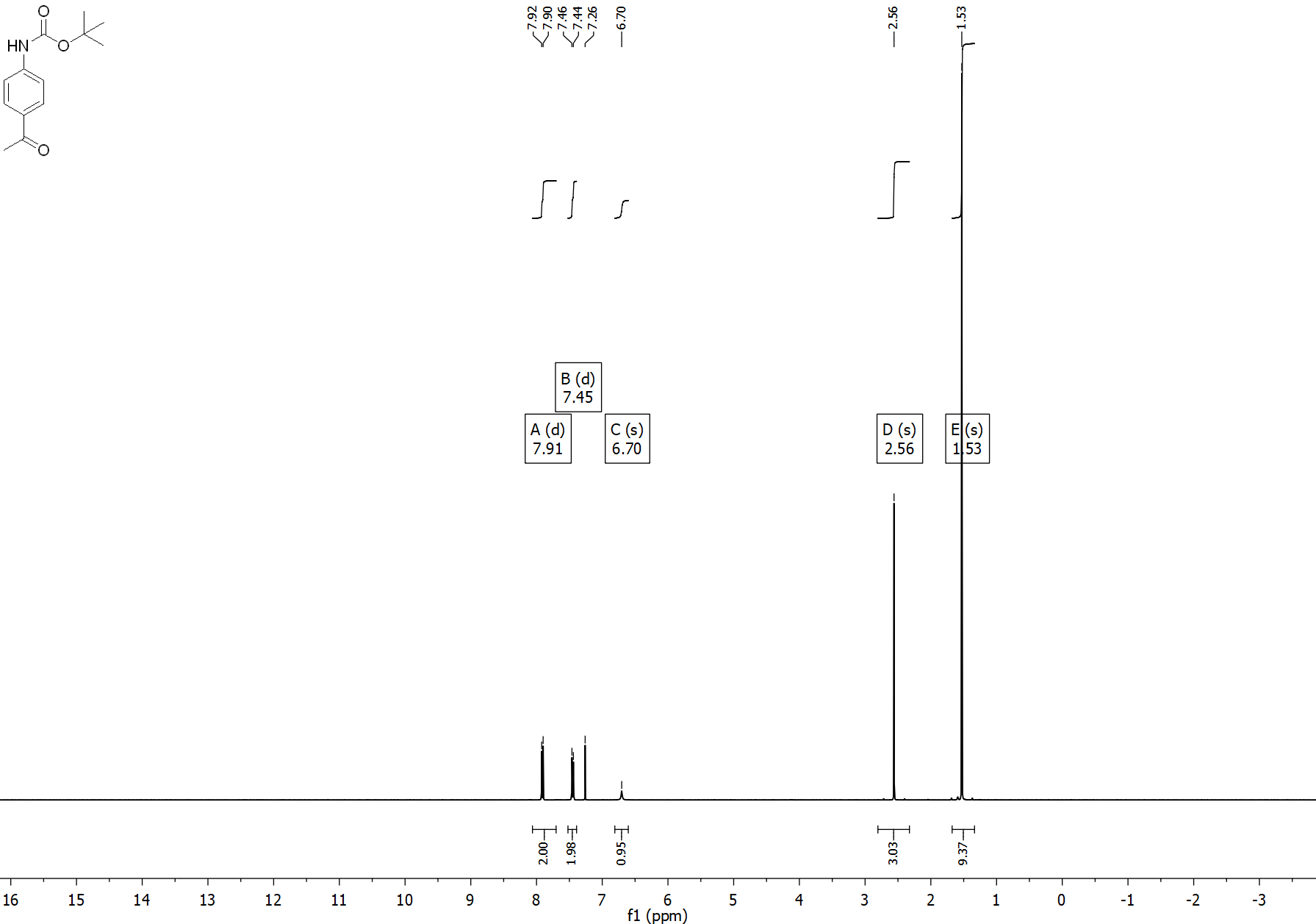


**Figure 12.** ^13^C-NMR spectra of compound **7**.


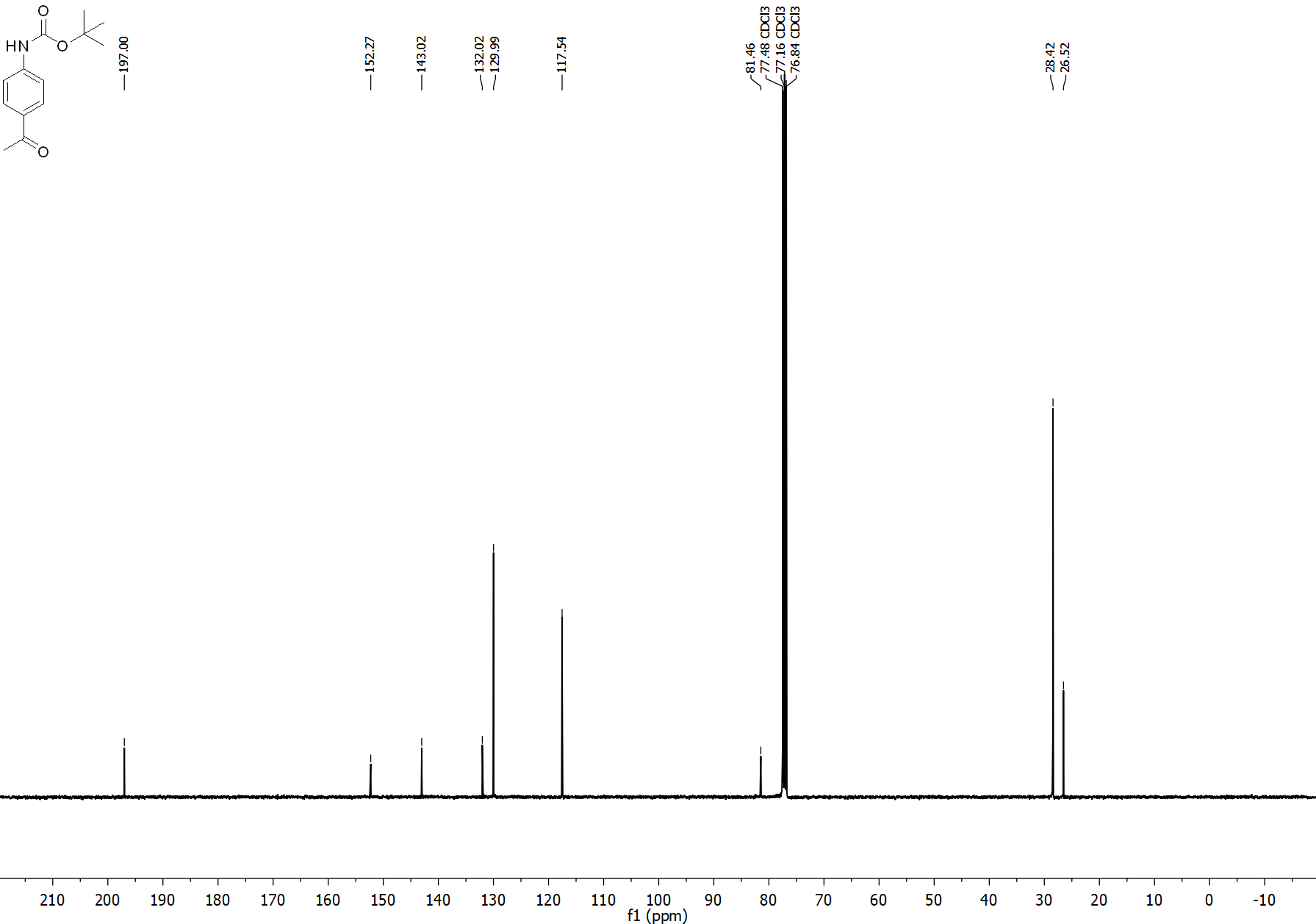
**Figure 13.** ^13^C-NMR spectra of compound **7**.


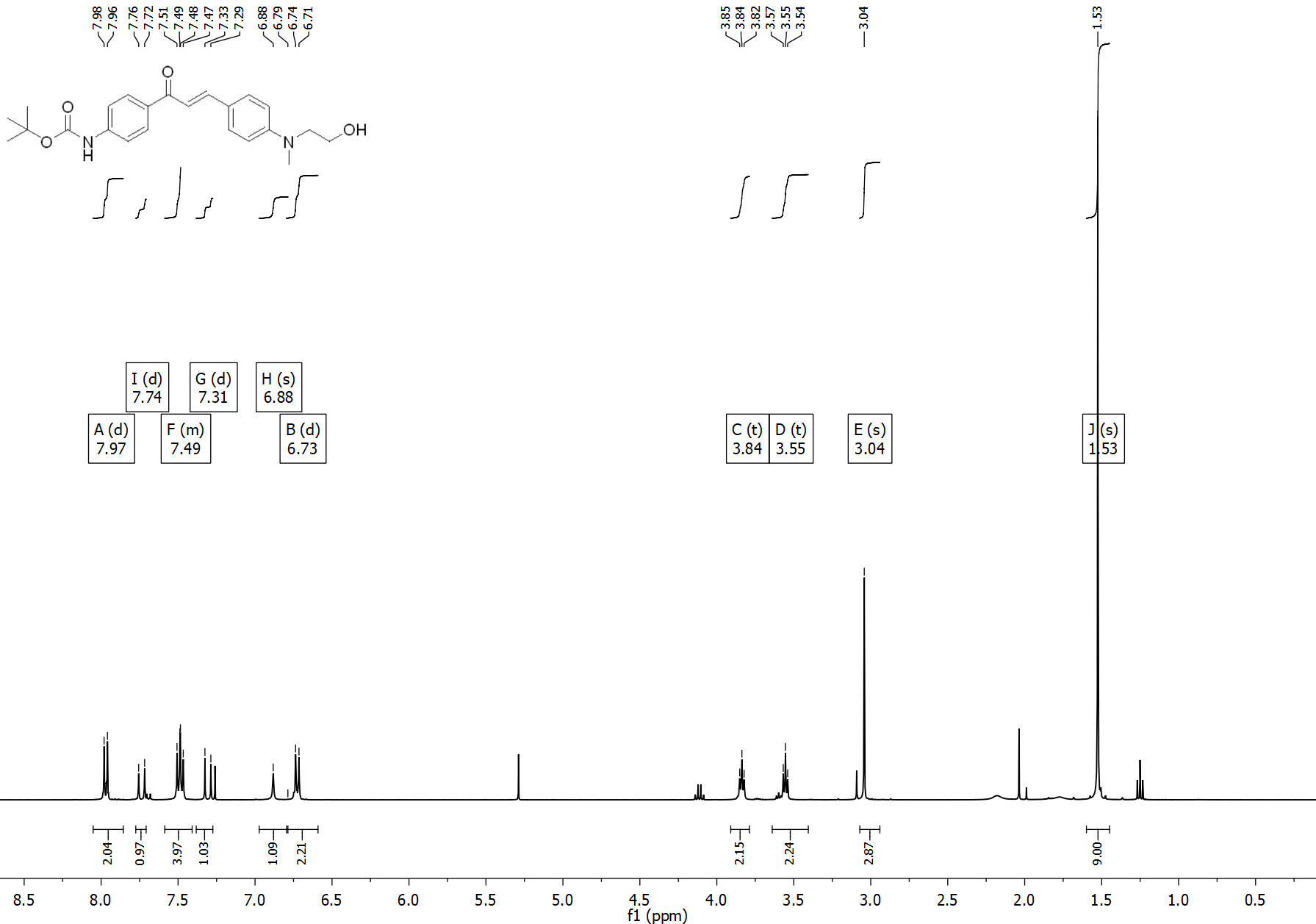
 **Figure 14.** ^1^H-NMR spectra of compound **8**.


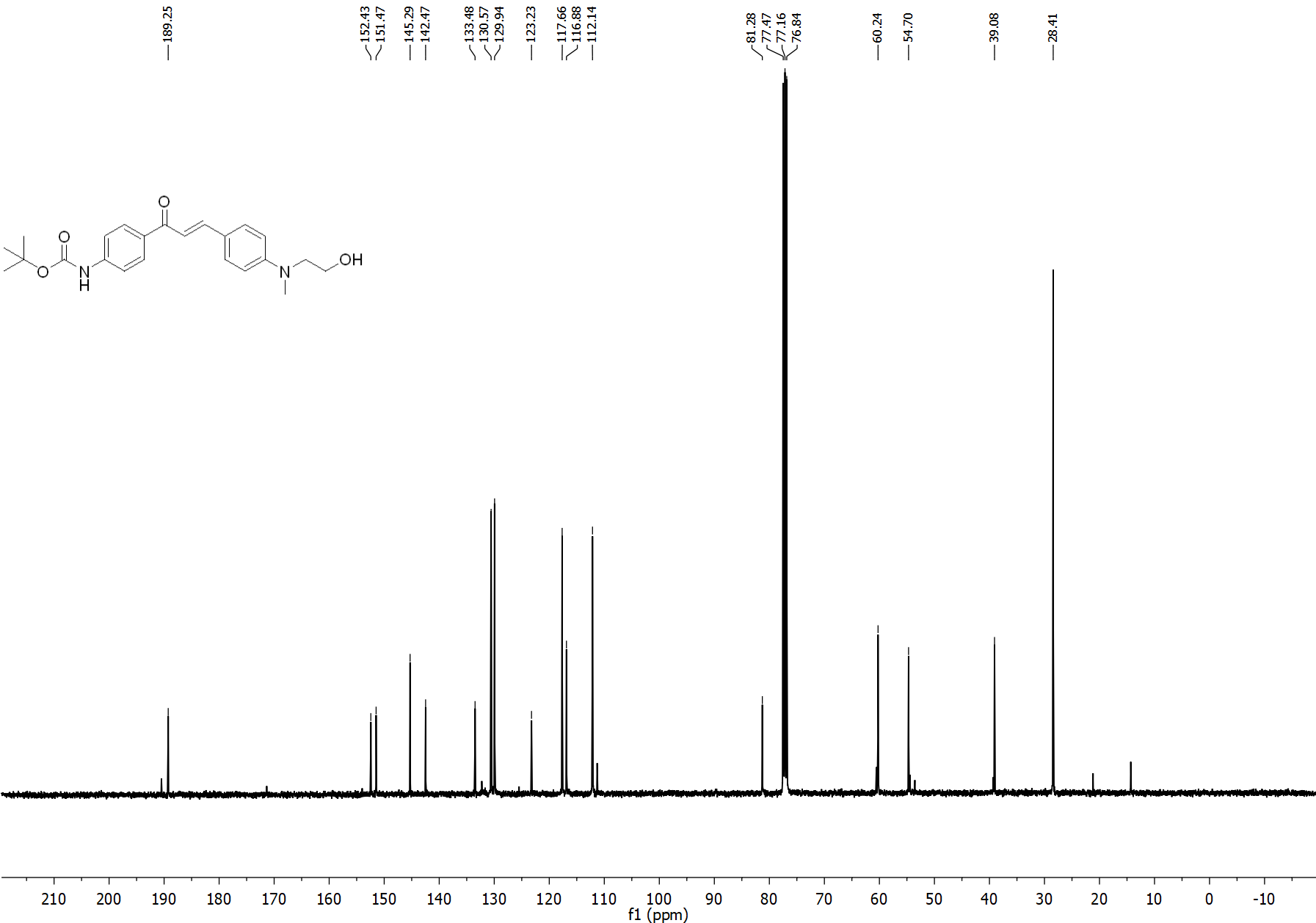


**Figure 15.** ^13^C-NMR spectra of compound **8**.


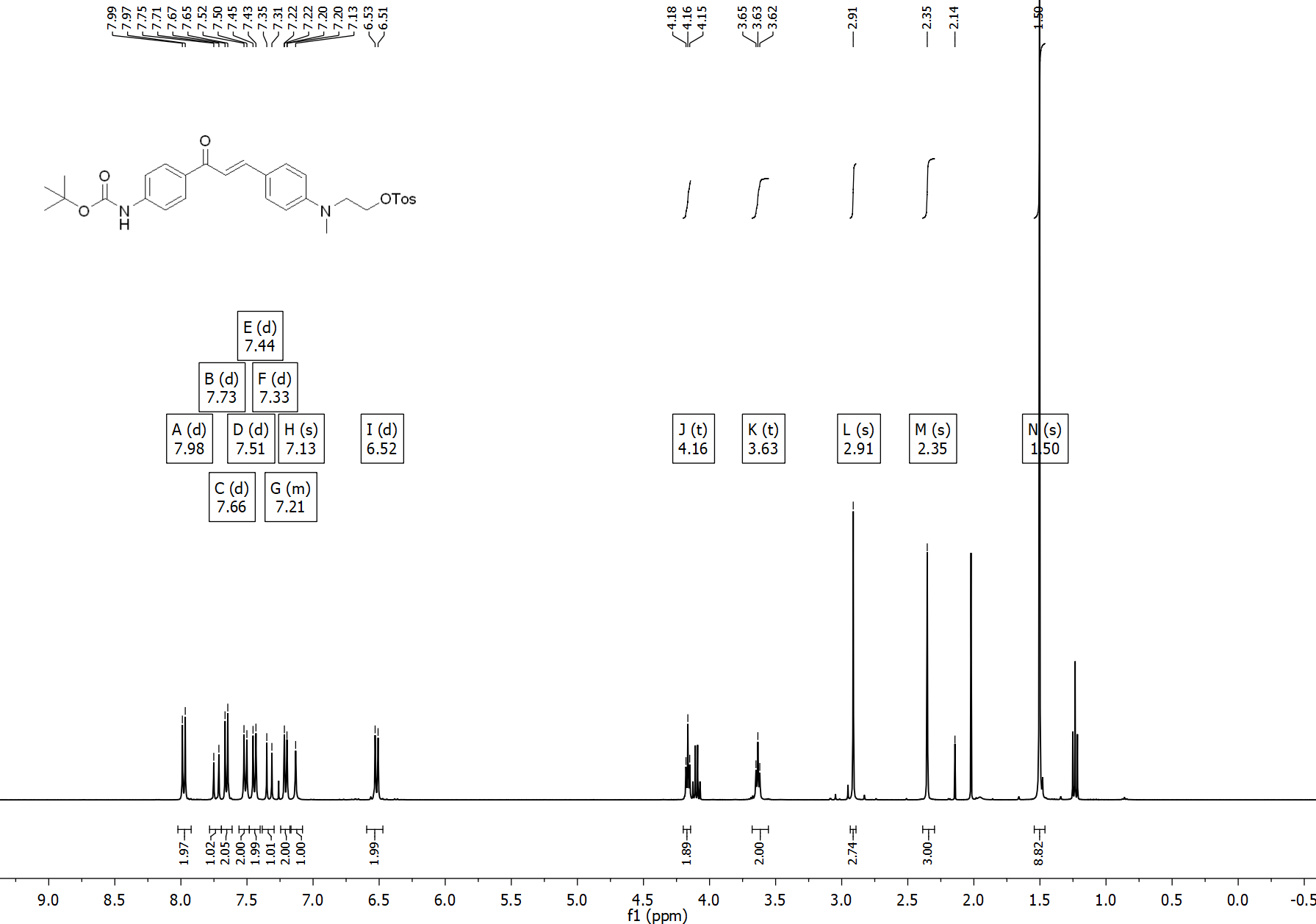


**Figure 16.** ^13^C-NMR spectra of compound **9**.


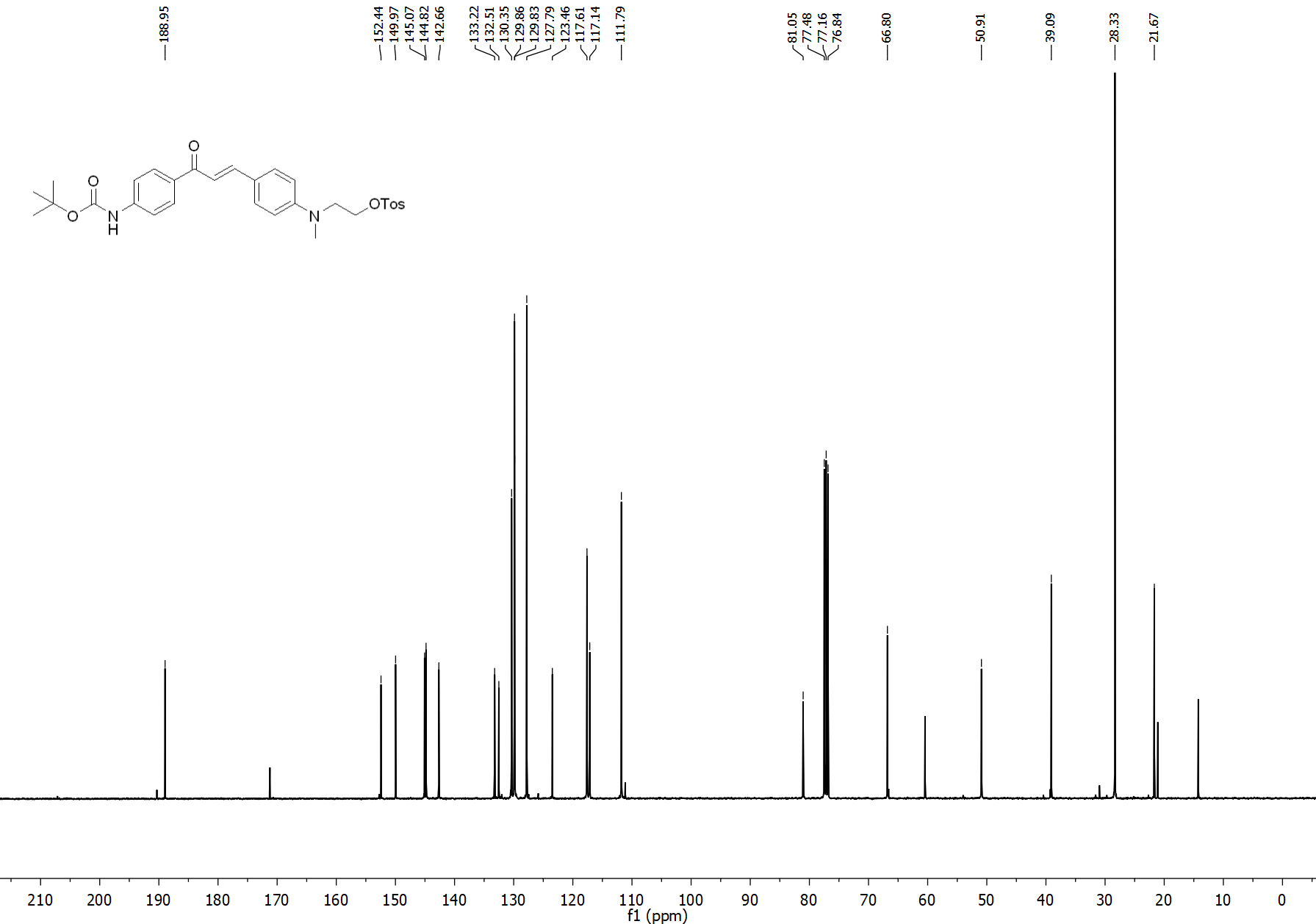
**Figure 17.** ^13^C-NMR spectra of compound **9**.

1. **UV-Vis/Fluorescence Spectroscopy**

**A B**

Em = 550 nm

Ex = 420 nm

**Figure 18. A**) UV-Vis Excitation λ_ex_ = 420 nm, **B**) Fluorescence Emission λ_em_ = 550 nm, spectra for compound **5**.

1. **Radiochemistry**

[^18^F]Fluoride was produced by a GE PETtrace cyclotron by 16 MeV irradiation of enriched [^18^O]H_2_O target, supplied by Alliance Medical Radiopharmacy Ltd (Warwick, UK). Automated radiosynthesies were performed using the GE FASTlab^™^ automated synthesis module (GE Healthcare Life Sciences, Amersham, UK). Solid phase extraction (SPE) cartridges were purchased from Waters (Elstree, Hertfordshire, UK) and used according to the manufacturers recommended guidelines.


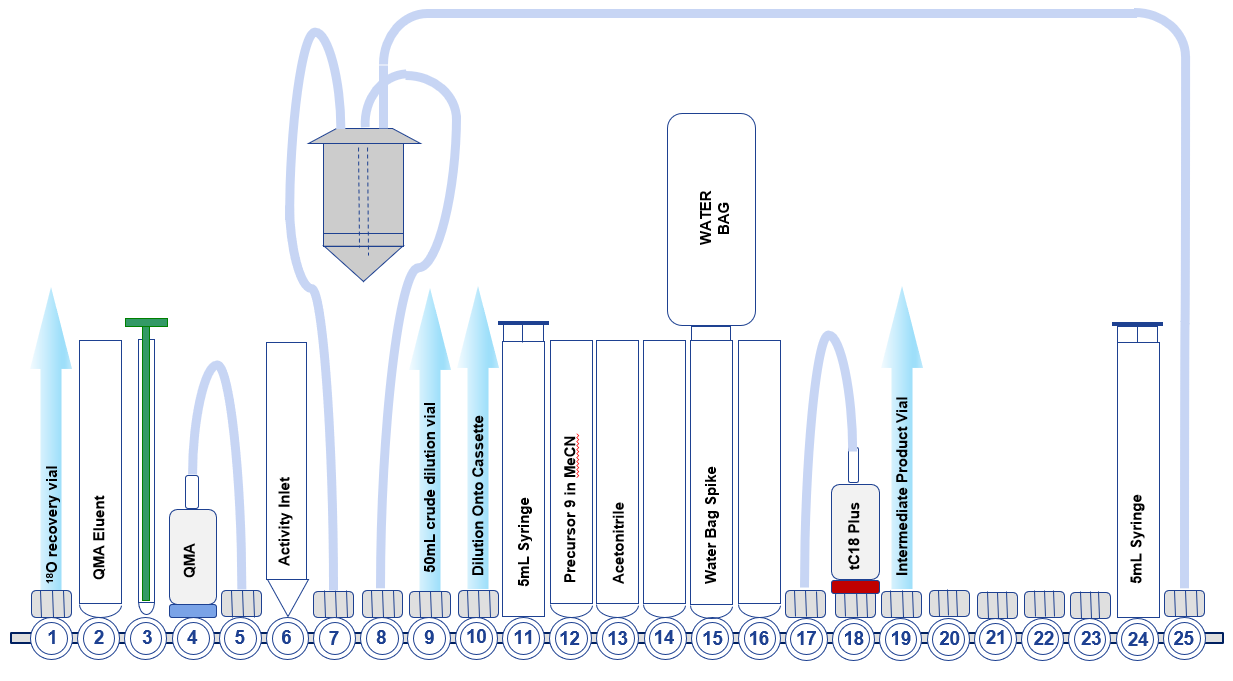


**Figure 19.** Graphical representation of the GE FASTLab™ cassette setup used for the radiosynthesis of compound **[^18^F]5**.

**Table 1.** Reagent positioning for the GE FASTLab™ cassette shown in Figure 19.

| Position |  |  | Position |  |
| --- | --- | --- | --- | --- |
| 1 | ^18^O water recovery vial |  | 14 | Empty |
| 2 | QMA Eluent (K_222_/KHCO_3_) |  | 15 | Water Bag |
| 3 | 1 mL syringe |  | 16 | Empty |
| 4 | QMA SPE cartridge |  | 17 | tC18 Plus SPE cartridge |
| 5 |  |  | 18 |  |
| 6 | ^18^F activity inlet |  | 19 | Product Vial |
| 7 | Reactor vessel |  | 20 | Empty |
| 8 |  |  | 21 |  |
| 9 | Crude dilution vial (50 mL H_2_O) |  | 22 |  |
| 10 | Return of crude dilution to cassette |  | 23 |  |
| 11 | 5 mL syringe |  | 24 | 5 mL syringe |
| 12 | Precursor **9** (1.6 mg) in MeCN (1.2 mL) |  | 25 | Reactor vessel |
| 13 | MeCN 2 mL |  |  |  |

1. **HPLC Chromatograms**


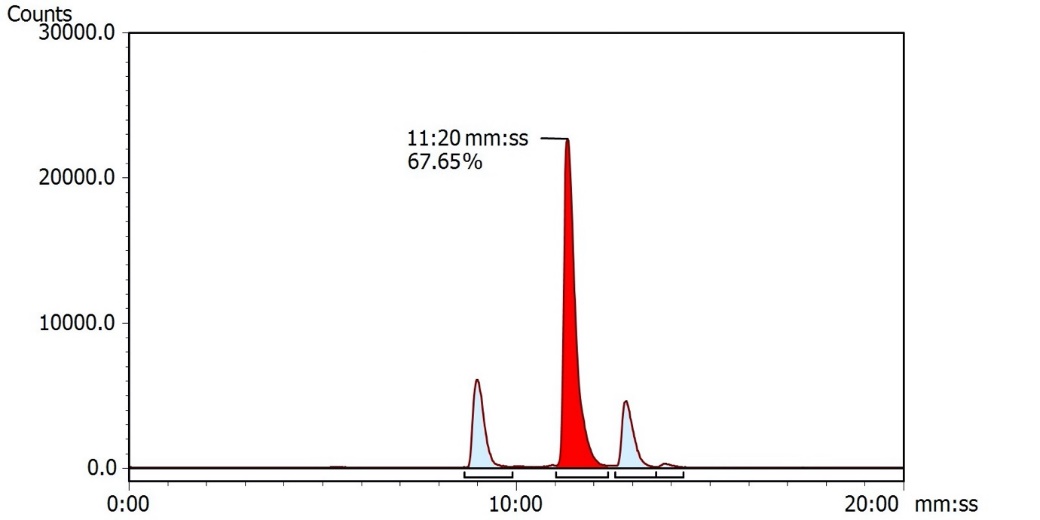


**Figure 20.** Representative radio-HPLC chromatogram of crude **[^18^F]10** after SPE purification.


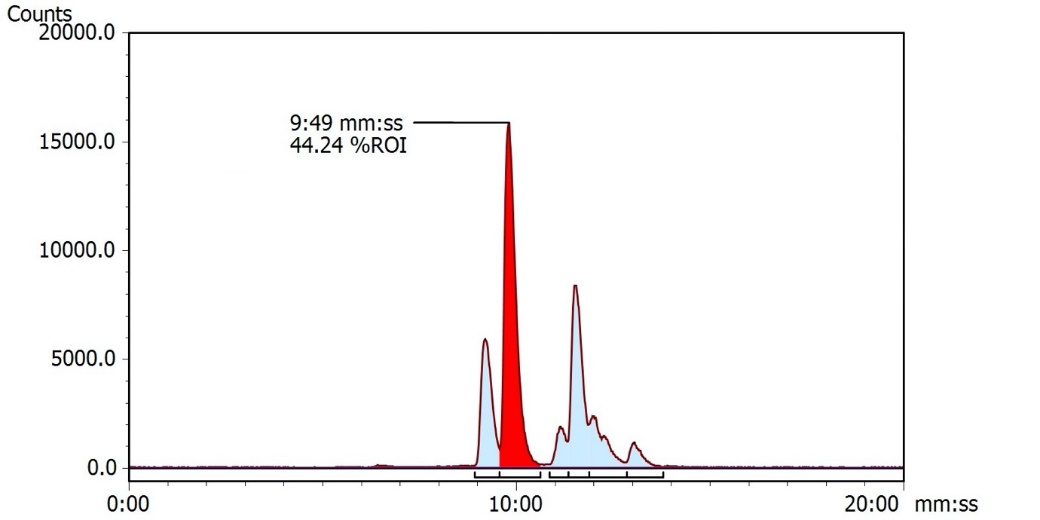


**Figure 21.** Representative radio-HPLC chromatogram of **[^18^F]4** in the crude reaction mixture.

**
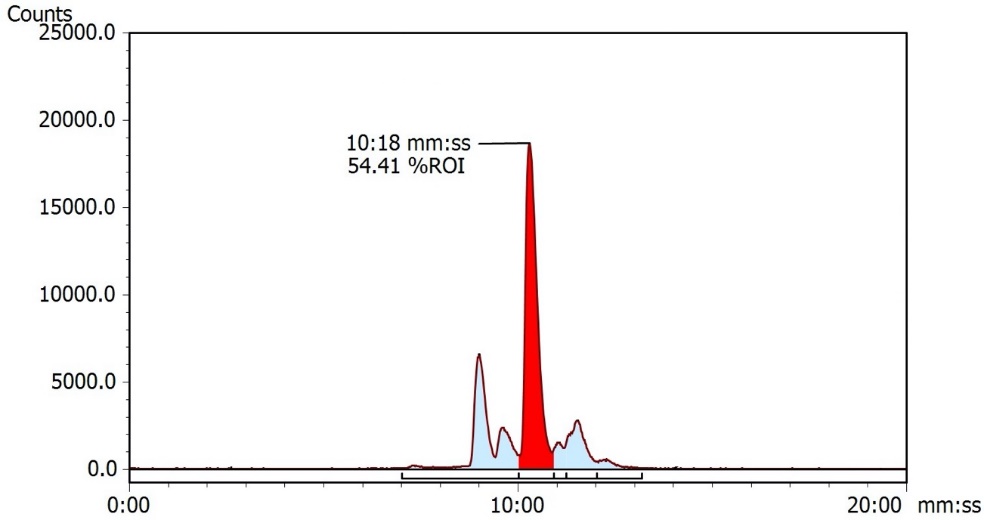
**

**Figure 22.** Representative radio-HPLC chromatogram of **[^18^F]5** in the crude reaction mixture.

**A**


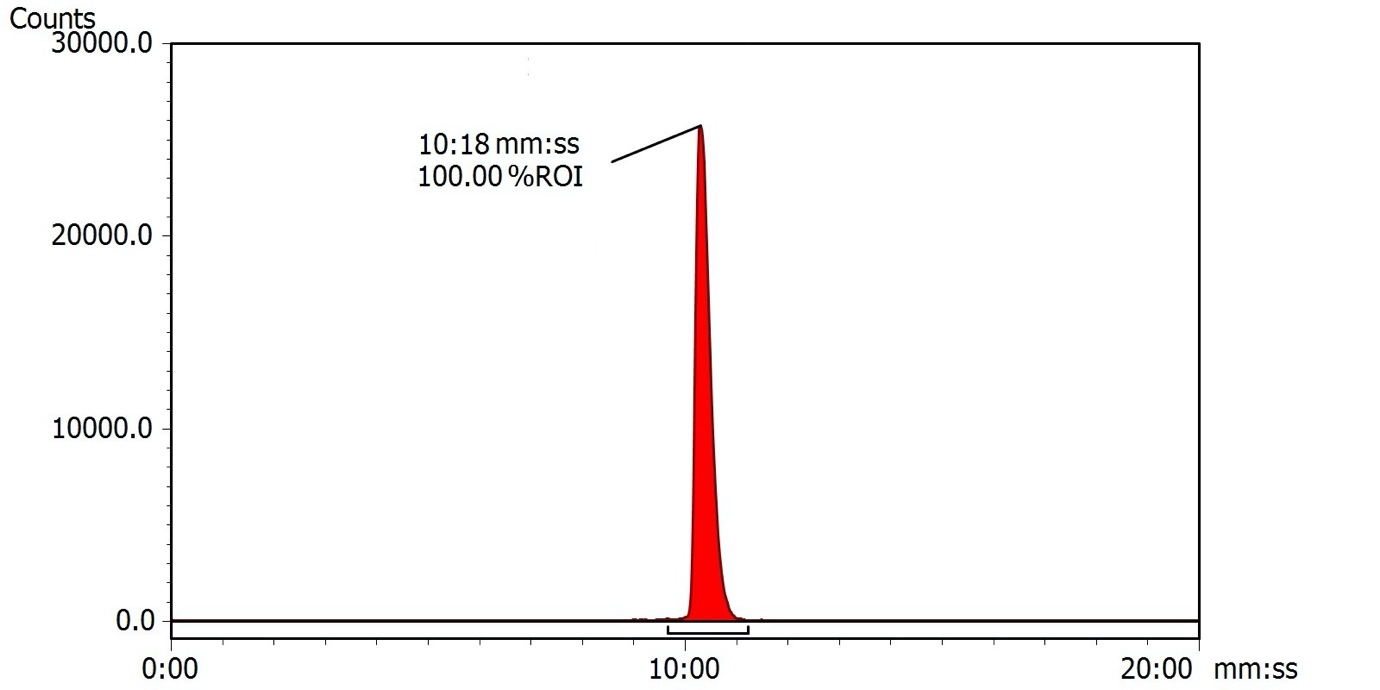


**B**


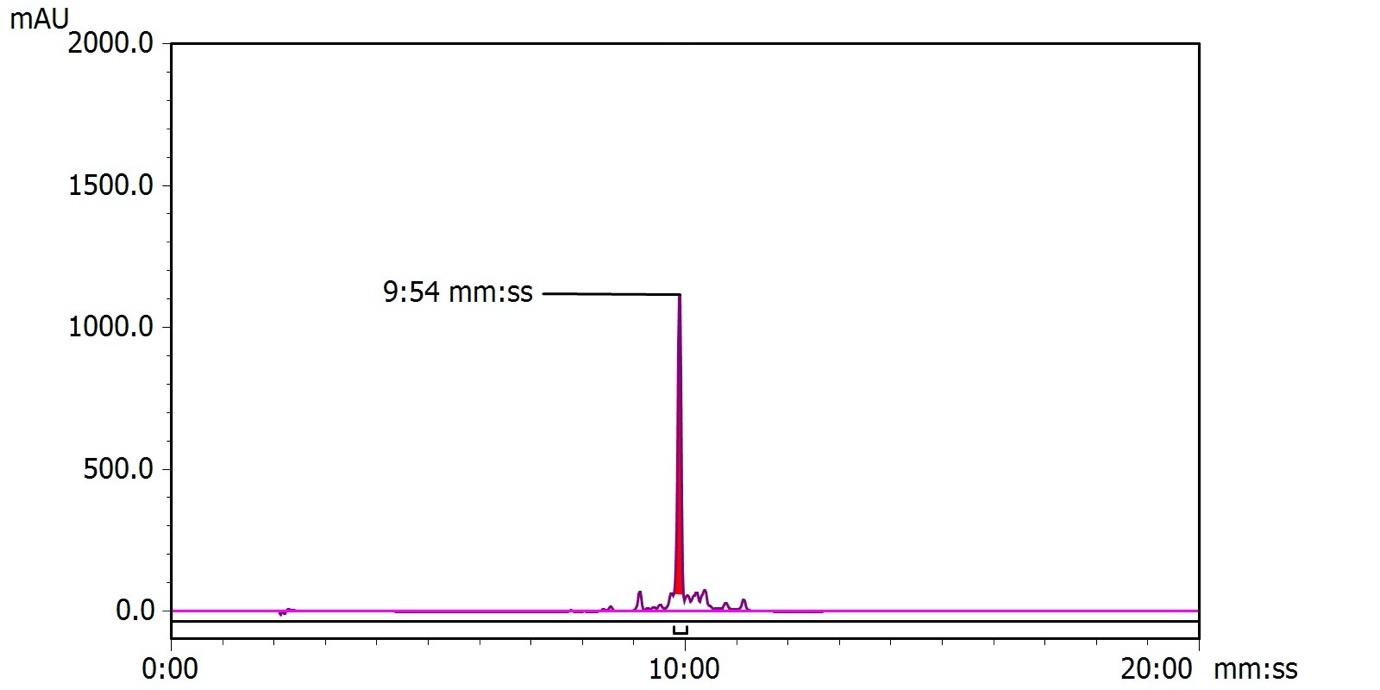


**Figure 23. A)** Representative radio-HPLC chromatogram and **B)** UV-HPLC chromatogram (λ = 254 nm) of **[^18^F]5** after final the semi-preparative purification and reformulation into EtOH with co-injection of **5**.
